# A chronological framework for atherosclerosis: from environmental pressure to clinical disease, with vascular aging as a cross-cutting dimension

**DOI:** 10.3389/fcvm.2026.1941915

**Published:** 2026-10-07

**Authors:** Jorge E. Montoya-Pérez, Juan R. Serna-Garza, Abraham E. Gracia-Ramos

**Affiliations:** 1 Centro Integral de Riesgo Cardiovascular, Envejecimiento Vascular y Sueño (CIRCEVS), Cuauhtémoc, Chihuahua, México; 2 Práctica Privada de Cardiología, Playa del Carmen, Quintana Roo, México; 3 Departamento de Medicina Interna, Hospital General, Centro Médico Nacional “La Raza”, IMSS, Ciudad de México, México

**Keywords:** apolipoprotein B, atherosclerosis, cardiovascular risk stratification, disease staging, insulin resistance, lipoprotein(a), vascular aging

## Abstract

Atherosclerosis is a multiphase disease, yet contemporary cardiovascular risk stratification relies predominantly on calculators that estimate event probability within a limited pathophysiological window. Framingham, SCORE2, GLOBORISK and PREVENT capture lipid burden, metabolic parameters, and renal function, but they do not organize the chronological sequence of atherogenesis, assign measurable biomarkers to each stage, or integrate vascular aging. We propose CASCADE (Chronological Atherosclerosis Staging: Connecting Aging, Disease, and Environment), a conceptual framework that applies disease staging to atherosclerosis, organizing it into seven pathophysiological phases (F1–F7) preceded by an upstream environmental and behavioral pressure layer (F0), and spanning the trajectory from adaptive metabolic response to clinical cardiovascular disease. Each phase is mapped to a proposed dominant biomarker — from insulin resistance indices through apolipoprotein B, lipoprotein(a), and coronary artery calcium — and to a reversibility zone (largely reversible, transition, or stabilization only). Vascular aging — characterized by arterial stiffness, cellular senescence, epigenetic drift, and clonal hematopoiesis — traverses all phases as a modifiable cross-cutting dimension, with specific biological clocks applicable at each stage. The framework is developed through narrative synthesis of mechanistic, epidemiological, and clinical trial evidence, integrating current international guidelines and emerging evidence on biological aging and environmental determinants of cardiovascular risk. CASCADE complements existing risk calculators by providing chronological context for the upstream phases (F0–F3), where conventional tools offer limited guidance and the potential for disease modification is greatest. It is proposed as a hypothesis-generating framework, not a validated scoring system, and invites prospective evaluation of whether individuals traverse identifiable, modifiable phases of atherosclerotic disease — and whether integrating vascular aging measurement at each stage improves risk assessment and therapeutic decision-making.

## Introduction

Atherosclerosis remains the leading cause of cardiovascular morbidity and mortality worldwide, yet its clinical assessment continues to rely predominantly on risk calculators that capture only a fraction of its biological complexity. The Framingham Risk Score, developed from a largely homogeneous North American cohort, estimates 10-year event probability using age, sex, blood pressure, cholesterol, diabetes, and smoking status — variables that map primarily to the intermediate and late phases of atherogenesis (1). The European SCORE2 system similarly operates within the window of established lipid burden and clinical risk factors, without addressing earlier metabolic or vascular processes (2). More recently, the American Heart Association/American College of Cardiology (AHA/ACC) PREVENT equations expanded this scope by incorporating kidney function and metabolic markers, representing a meaningful advance toward multisystem assessment (3). However, PREVENT, like its predecessors, functions as a statistical prediction tool rather than a framework that organizes the pathophysiological sequence of atherosclerotic disease.

The European Atherosclerosis Society has recently proposed clinical staging of metabolic disorders as an approach to guide management of their cardiovascular sequelae (4), and a European Society of Cardiology (ESC)/ACC/AHA/World Heart Federation joint statement has formalized environmental risk factors — including air pollution, noise, extreme temperatures, and plastic pollution — as determinants of cardiovascular disease requiring integrated management (5). A parallel proposal in diabetes advocates replacing the binary term “prediabetes” with three chronological stages of type 2 diabetes, arguing that cardiovascular damage begins before diagnostic thresholds are met and that staging — as previously demonstrated for hypertension and type 1 diabetes — enables regulatory pathways, diagnostic coding, and earlier therapeutic intervention (6). Most consequentially, a recent Lancet Commission on rethinking coronary artery disease — developed by 25 international authors across 357 references — has proposed reclassifying the condition as Atherosclerotic Coronary Artery Disease (ACAD) and redirecting clinical attention from ischemia to the lifelong detection of atheroma, modeling that elimination of behavioral and metabolic risk factors by 2050 would reduce ACAD deaths by 82.1% (approximately 8.7 million lives annually) (7). These developments signal a growing recognition that atherosclerotic risk extends far beyond traditional lipid-centric assessment, yet no published framework translates this emerging paradigm into an operational architecture — chronological sequencing, phase-specific biomarkers, and stage-dependent therapeutic decisions.

This distinction matters because atherosclerosis is not a single-event phenomenon. Decades of mechanistic research have revealed a cascade that begins well before lipoprotein retention in the arterial wall — the process captured by the response-to-retention hypothesis (8, 9). Upstream of retention, insulin resistance drives metabolic and vascular dysfunction through impaired PI3 K/Akt and compensatory MAPK/endothelin-1 (ET-1) signaling in endothelial cells (10). Vascular stiffening, measurable by pulse wave velocity (PWV), precedes and accelerates plaque formation. Environmental and behavioral pressures — including ultra-processed food consumption, air pollution, and psychosocial stress — create the cardiometabolic substrate upon which this cascade unfolds (5, 11, 12). Meanwhile, vascular aging — characterized by progressive arterial stiffness, endothelial dysfunction, cellular senescence, and extracellular matrix remodeling — modulates every stage of this process (13, 14). A state-of-the-art review from the ESC Council on Basic Cardiovascular Science has catalogued the emerging molecular and cellular pathways linking metabolic disorders to cardiovascular pathology, underscoring the need for integrative frameworks (15). Similarly, a comprehensive review of cardiovascular biomarkers for primary prevention stratification has highlighted that conventional risk factors have moderate discrimination and high residual risk, reinforcing the rationale for expanded biomarker-guided assessment (16).

Despite this growing understanding, no published framework integrates these observations into a chronologically organized model that spans from primordial environmental exposures through subclinical disease, while assigning measurable biomarkers to each stage and incorporating vascular aging as a cross-cutting dimension. Existing risk tools map, in our interpretation, primarily to F4 and F7 in the model proposed here, without a sequential organizational logic. The response-to-retention hypothesis explains a molecular mechanism but does not extend upstream to metabolic and environmental origins or downstream to plaque vulnerability and clinical manifestation. Vascular aging research has accelerated markedly — with epigenetic clocks, proteomic aging signatures, and clinical vascular age metrics now available (13) — but this dimension remains disconnected from conventional atherosclerosis staging.

The 2026 ACC/AHA Multisociety Guideline on the Management of Dyslipidemia has formalized several elements that align with a staged approach to atherosclerosis. It established universal once-in-a-lifetime lipoprotein(a) [Lp(a)] measurement as a Class I recommendation, introduced apolipoprotein B (apoB) as a confirmatory measurement (Class IIa) in patients with atherosclerotic cardiovascular disease (ASCVD), cardiovascular-kidney-metabolic (CKM) syndrome, diabetes, or elevated triglycerides, and replaced the Pooled Cohort Equations with the PREVENT risk calculator, which explicitly incorporates kidney function, metabolic markers, and social determinants of health. The guideline also defined a four-tiered coronary artery calcium ladder (1–99, 100–299, 300–999, ≥1000) with stratum-specific low-density lipoprotein cholesterol (LDL-C) and apoB targets, recognized high-sensitivity C-reactive protein (hs-CRP) ≥2 mg/L on two occasions as a trigger for high-intensity statin therapy in borderline-risk patients, and acknowledged CKM syndrome — with albuminuria as a defining component — as a condition that independently escalates cardiovascular risk (17). These guideline changes validate several of the architectural decisions underlying CASCADE — apoB primacy at F4, universal Lp(a) testing at F5, albuminuria as a standalone predictor of renal and vascular disease at F3, and graduated intensity of intervention based on subclinical atherosclerosis at F6 — but they do not propose a chronological architecture integrating environmental pressure, metabolic response, and vascular aging into a single operational model.

CASCADE (Chronological Atherosclerosis Staging: Connecting Aging, Disease, and Environment) is proposed as a conceptual framework that addresses this gap. It organizes atherosclerosis into seven pathophysiological phases (F1–F7), preceded by an upstream environmental and behavioral pressure layer (F0). Each phase is mapped to a proposed dominant biomarker and a set of supporting variables, while vascular aging traverses all phases as a modifiable cross-cutting dimension — that is, a dimension that cuts across all phases rather than progressing along the temporal sequence. The framework does not replace existing risk calculators but rather provides a chronological architecture within which they can be contextualized.

This narrative review presents the scientific rationale for CASCADE, examines the evidence supporting each phase and its biomarker assignments, discusses vascular aging as a cross-cutting dimension, and acknowledges the limitations and testable hypotheses that the framework generates. The intent is not to present a validated scoring system but to propose an organizational model that invites empirical evaluation by the research community.

## Methods

This article is a narrative review, and its search strategy is summarized here for transparency. The framework was conceived during 2025, with two publications acting as conceptual seeds (10, 18). Between January and August 2026, the author conducted a manual literature search in PubMed, Google Scholar, and the Nature portfolio journals, complemented by a personal full-text library of approximately 390 articles assembled over the same period. Search terms corresponded to the keywords of this article. Publications in English and Spanish were considered. Consistent with the narrative design, no formal inclusion or exclusion criteria were applied: priority was given to guidelines, consensus documents, and studies published from 2010 onward — with emphasis on the most recent evidence — while earlier foundational works were retained where they anchor a concept, such as the response-to-retention hypothesis (8) and the original Framingham risk equations (1). Regional sources reviewed but excluded as out of scope included the Mexican GREHTA study and Mexican national clinical guidelines. The study design of each cited reference, classified into six descriptive levels (from randomized trials and meta-analyses to preprints and commentaries) and tabulated by CASCADE phase, is provided in Supplementary Table S1. The use of generative artificial intelligence was limited to what is declared in the Acknowledgments.

## Section 1: the chronological model

CASCADE proposes seven pathophysiological phases of atherosclerosis (F1–F7), preceded by an upstream environmental and behavioral layer (F0) that constitutes the substrate of cumulative exposure rather than a biological phase of the individual. The chronological framework therefore comprises eight measurable tiers — one environmental and seven biological — each with assigned biomarkers (Figure 1). For consistency with the chronological architecture, the environmental tier is labeled F0; the term seven phases refers throughout this paper to the biological response cascade (F1–F7).

**Figure 1:**
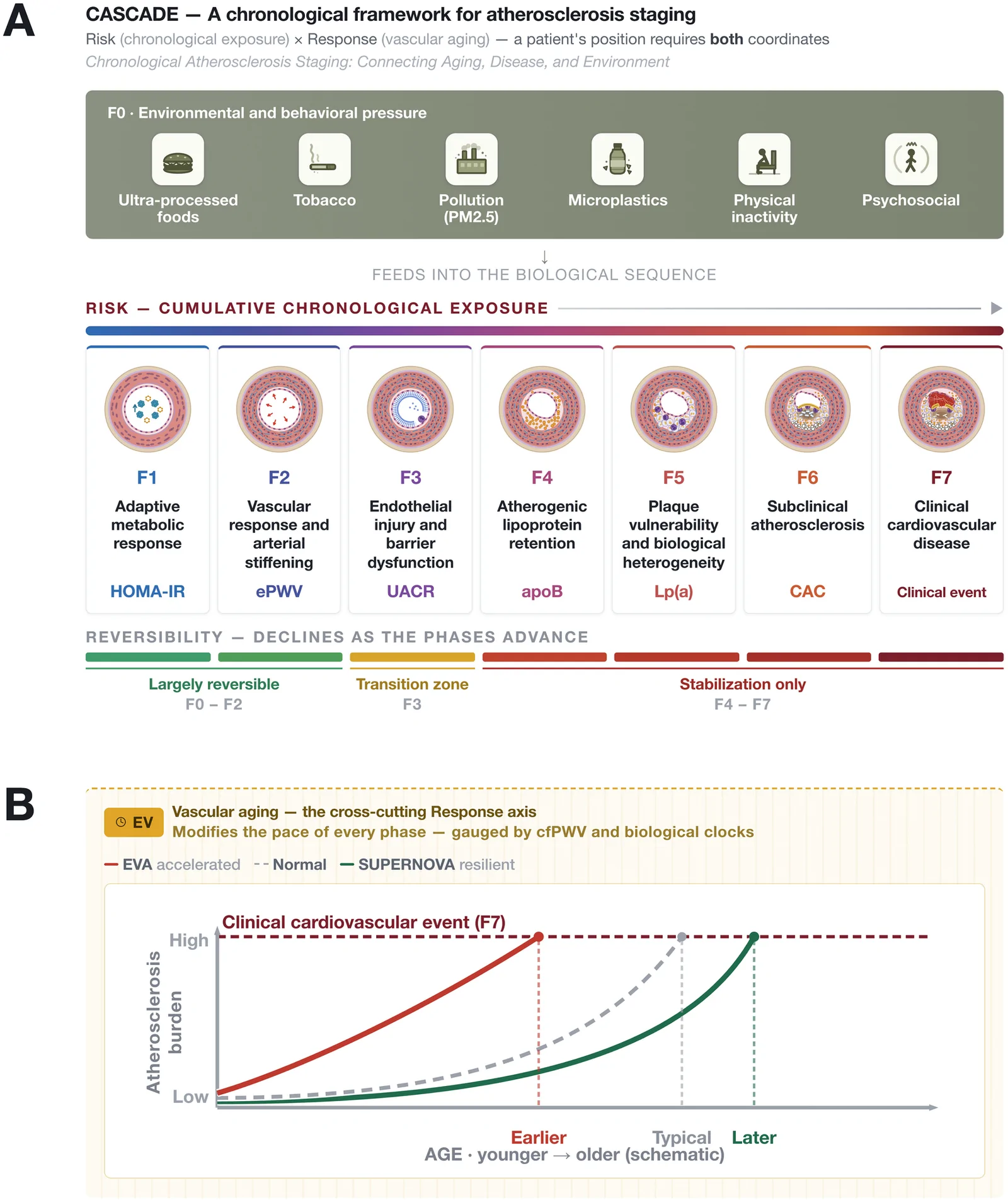
Central illustration — CASCADE: chronological atherosclerosis staging with vascular aging as a cross-cutting dimension. **(A)** The framework organizes atherosclerosis along a horizontal Risk axis representing cumulative chronological exposure. An environmental and behavioral pressure layer (F0 — ultra-processed foods, tobacco, PM2.5 air pollution, microplastics, physical inactivity, and psychosocial stress) feeds into a biological sequence of seven phases: F1, adaptive metabolic response (HOMA-IR); F2, vascular response and arterial stiffening (ePWV); F3, endothelial injury and barrier dysfunction (UACR); F4, atherogenic lipoprotein retention (apoB); F5, plaque vulnerability and biological heterogeneity [Lp**(a)**]; F6, subclinical atherosclerosis (CAC); and F7, clinical cardiovascular disease (clinical event). Each phase is anchored by a proposed dominant biomarker. A reversibility gradient runs beneath the sequence: largely reversible (F0–F2), a transition zone (F3), and stabilization only (F4–F7). **(B)** Vascular aging is the cross-cutting Response axis that modifies the pace at which a patient traverses the phases, gauged by carotid-femoral pulse wave velocity (cfPWV) and biological clocks. For a comparable cumulative exposure, an accelerated phenotype (early vascular aging, EVA) reaches the clinical event (F7) at a younger age, a resilient phenotype (SUPERNOVA) reaches it later, and a normal trajectory lies between them. A patient's position in the framework therefore requires both coordinates: Risk (chronological exposure) and Response (vascular aging). Panel labels **(A)** and **(B)** refer to the two figure files supplied separately. The illustration is schematic and conceptual; the curves in **(B)** do not represent empirical data. apoB, apolipoprotein B; CAC, coronary artery calcium; ePWV, estimated pulse wave velocity; HOMA-IR, Homeostatic Model Assessment of insulin resistance; Lp**(a)**, lipoprotein**(a)**; PM2.5, fine particulate matter; UACR, urine albumin-to-creatinine ratio.

### F0: environmental and behavioral pressure

The cascade begins upstream of any measurable biological response: in the environmental, nutritional, and behavioral exposures that create the cardiometabolic substrate for disease. The 2026 AHA dietary guidelines identify ultra-processed food consumption as a driver of cardiovascular risk through mechanisms that extend beyond caloric excess, including disruption of satiety signaling, alterations in the gut microbiome, and promotion of systemic inflammation (11). Air pollution — particularly fine particulate matter (PM2.5) — increases coronary artery disease risk by 28% per 10 µg/m^3^ increment, operating through cardiometabolic mediators including systolic blood pressure (13% mediation) and LDL-C (9% mediation), with amplification in individuals with high genetic susceptibility (19). Psychosocial stressors also contribute: social isolation and loneliness have been independently associated with incident cardiovascular disease in older women (12), and a translational pilot study links them to endothelial dysfunction, with the combination of circulating epinephrine and tumor necrosis factor α (TNFα) associating with reduced endothelial VE-cadherin expression and, *in vitro*, impairing endothelial barrier integrity through partly JAK/STAT-dependent signaling (20).

Beyond dietary composition and air quality, additional environmental inputs amplify the cardiometabolic substrate of F0. Gut-derived metabolites contribute mechanistically: imidazole propionate (ImP) activates mTOR signaling and promotes vascular inflammation (21). Environmental contaminants are detectable within human atheromas themselves: in a prospective multicenter study of patients undergoing carotid endarterectomy, polyethylene was found in 58.4% of excised plaques, and patients with microplastics present in their atheromas had a significantly higher risk of the composite cardiovascular endpoint (HR 4.53, 95% CI: 2.00–10.27) (18).

The biological impact of upstream exposures extends beyond cardiovascular endpoints — and is both quantifiable and modifiable. A randomized trial of home-delivered Dietary Approaches to Stop Hypertension (DASH)-patterned groceries reduced systolic blood pressure by 5 mmHg and LDL-C by 7 mg/dL in already-treated hypertensive adults, demonstrating that dietary intervention at F0 is additive to pharmacotherapy, not redundant (22). At the population level, suboptimal diet was responsible for a 42% increase in absolute ischemic heart disease mortality over 33 years across 204 countries (even as age-standardized rates declined), driven primarily by insufficient intake of nuts, seeds, whole grains, and fruits rather than excess of any single dietary component (23). An exposome-phenome atlas mapping 619 exposures to 305 phenotypes in NHANES demonstrated that 20 aggregated exposomic factors explained variance comparable to approximately one million genomic single-nucleotide polymorphisms (SNPs), with cardiometabolic phenotypes — triglycerides, glycated hemoglobin (HbA1c), body mass index (BMI) — as targets of maximum predictive yield (24). In a global study of 18,701 participants across 34 countries with multimodal neuroimaging, aggregate exposomic models — encompassing physical and social environmental factors — explained up to 15.5 times more variance in brain aging than individual factors alone, with total exposomic burden conferring odds ratios of 3.3–9.1 for accelerated aging that exceeded the effect of established clinical diagnoses (25).

Temporal eating patterns represent a complementary upstream input. In an analysis of 21,006 adults who logged 2.65 million food and beverage records over 14 days via the myCircadianClock app, the median 95% eating window was 13 h 24 min, with the lowest decile at 10 h 54 min and the highest reaching 16 h, and fewer than 24% of users eating within a window shorter than 12 h (26).

F0 is the most modifiable layer of the framework: its exposures can be reduced or removed, although long-standing exposure may leave a biological imprint that does not fully resolve. Its proposed dominant biomarker is a composite ultra-processed food score (UPF-score), an operational proposal that requires external validation. Tobacco exposure (pack-years) and PM2.5 estimates serve as established alternative markers. The evidence presented above — spanning exposomic burden and psychosocial stress — suggests that F0 assessment may ultimately require a multidimensional composite integrating nutritional, environmental, psychosocial, and physical activity domains, rather than any single marker. The development and validation of such a composite represents a priority for future iterations of the framework.

### F1: adaptive metabolic response

Sustained environmental pressure triggers measurable cardiometabolic adaptations: visceral adiposity, compensatory hyperinsulinemia, metabolic insulin resistance, hypertriglyceridemia, and expansion of atherogenic lipoproteins. This phase does not yet involve structural vascular damage but creates the biological terrain upon which downstream processes unfold.

Insulin resistance, quantified by the Homeostatic Model Assessment (HOMA-IR), represents the proposed dominant biomarker for F1. The relationship between HOMA-IR and macrovascular and microvascular damage follows a nonlinear pattern with a saturation threshold at approximately HOMA-IR = 5, beyond which the incremental risk attenuates (27). This association was strongest in individuals under 50 years of age, supporting the rationale for early-phase assessment. Vascular insulin resistance — a distinct endothelial phenomenon characterized by impaired PI3 K/Akt signaling and compensatory MAPK/ET-1 activation — acts as a bridge between adaptive metabolic response (F1) and vascular response/arterial stiffening (F2) (10). A comprehensive review of the cellular mechanisms of insulin resistance in type 2 diabetes has further delineated the heterogeneity of insulin receptor signaling disruptions, emphasizing that hyperinsulinemia itself — through preserved lipogenic but impaired glucose-lowering pathways — contributes to atherogenic dyslipidemia (28). Type 2 diabetes has been reconceptualized as involving at least thirteen interrelated pathophysiological mechanisms — the “Tumultuous Thirteen” — with more than 70% of new cases attributable to suboptimal diet, reinforcing that the metabolic response of F1 is initiated by the environmental pressures of F0 (29).

Emerging evidence suggests bidirectional causality between metabolism and the vasculature. In murine models of overnutrition, endothelial FUNDC1 is upregulated and drives ET-1 overproduction that precedes and causes systemic insulin resistance, whereas its endothelial-specific deletion is protective (30). Similarly, endothelial spermidine deficiency activates RIPK1-mediated necroptosis, causing pancreatic microvascular damage that subsequently leads to insulin resistance in a progressive sequence from pancreas to kidney to adipose tissue (31). These findings suggest that what is measured as a “metabolic” phase may have vascular origins — a nuance that CASCADE acknowledges by placing metabolic and vascular responses in adjacent but separate phases.

Psychiatric comorbidity is increasingly recognized as a modulator of metabolic response: chronic depression and anxiety share bidirectional mechanisms with insulin resistance, inflammation, and mitochondrial dysfunction, contributing to 10–20 years of reduced life expectancy in psychiatric patients, predominantly from cardiovascular causes (32). This positions diagnosable mental health conditions within F1 as a metabolic modulator, distinct from the social and behavioral stressors of F0.

F1 is largely reversible at the level of its metabolic phenotype: visceral adiposity, HOMA-IR, and hyperinsulinemia respond to lifestyle and pharmacological intervention. Weight reduction with semaglutide reduced major adverse cardiovascular events (MACE) by 20% in the SELECT trial, with a prespecified analysis confirming consistent benefit across adiposity categories, supporting the modifiability of this phase (33). The psychiatric modulator component is modifiable rather than uniformly reversible: chronic depression and anxiety respond partially to integrated metabolic-psychiatric intervention, but residual symptoms and biological scars — including hypothalamic-pituitary-adrenal (HPA)-axis dysregulation and persistent low-grade inflammation — may persist even with optimal treatment. This asymmetry — largely reversible metabolic phenotype, partially reversible psychiatric modulator — is what positions diagnosable mental health conditions within F1 as a modulator rather than as a defining feature of the phase.

### F2: vascular response and arterial stiffening

Before lipoproteins are retained and plaque forms, the vasculature itself changes. Arterial stiffening — measurable by PWV — reflects structural and functional alterations in the vessel wall including elastin fragmentation, collagen deposition, and smooth muscle cell phenotypic switching. Estimated PWV (ePWV), derivable from age and blood pressure, is proposed as the dominant biomarker for this phase. ePWV is calculated from age and mean blood pressure using the equation derived from the Reference Values for Arterial Stiffness Collaboration (34) and validated as a prognostic marker in an independent population-based cohort (35).

Vascular insulin resistance drives this process directly: the imbalance between vasodilatory (PI3 K/Akt/nitric oxide [NO]) and vasoconstrictive (MAPK/ERK/ET-1) insulin signaling pathways reduces NO bioavailability and increases vascular tone (10). In human cohorts, endothelial alterations — reflected in circulating markers of glycocalyx integrity and adhesion molecule expression — correlate with diastolic dysfunction and adverse cardiovascular outcomes, suggesting that vascular stiffening has measurable consequences even before atherosclerotic plaque develops (36).

The temporal primacy of vascular dysfunction over metabolic dysfunction under conditions of caloric excess has been demonstrated experimentally: in high-fat diet models, endothelial dysfunction and impaired vasodilation can precede overt systemic insulin resistance (30). This observation supports CASCADE's architectural decision to position vascular response and arterial stiffening (F2) as potentially preceding, rather than merely accompanying, adaptive metabolic response (F1) under certain conditions — while acknowledging that in many patients these phases are concurrent.

F2 is largely reversible in its functional component: endothelial tone and smooth muscle reactivity can improve with intervention — through blood pressure control, regular aerobic exercise, dietary sodium reduction, and smoking cessation — but structural changes (elastin fragmentation) are largely irreversible.

### F3: endothelial injury and barrier dysfunction

F3 proposes that endothelial barrier dysfunction — reflected clinically by elevated urinary albumin-to-creatinine ratio (UACR) — represents a measurable window between vascular response/arterial stiffening (F2) and atherogenic lipoprotein retention (F4).

The rationale is as follows. The endothelial glycocalyx — a carbohydrate-rich layer lining the vascular lumen — regulates permeability, mechanotransduction, and leukocyte adhesion. In preclinical models, glycocalyx degradation, impaired NO-dependent vasodilation, increased endothelial permeability, and endothelial stiffening coincide temporally in the early pre-atherosclerotic phase (37, 38). Importantly, these events occur before organized plaque develops but the study did not demonstrate that glycocalyx damage precedes the other manifestations — they coincide. In human cohorts, UACR — a systemic proxy for endothelial integrity — predicts cardiovascular events across diabetic and non-diabetic populations (39), and its reduction by sodium-glucose cotransporter 2 (SGLT2) inhibitors and glucagon-like peptide-1 (GLP-1) receptor agonists correlates with improved cardiovascular outcomes (40, 41).

CASCADE acknowledges that the separation of endothelial injury and barrier dysfunction (F3) from atherogenic lipoprotein retention (F4) represents the framework's most debatable architectural decision. Two alternatives were formally considered. The first — merging both processes into a single endothelial-retention phase — would align with the response-to-retention hypothesis (8, 9), which treats subendothelial entry and retention as a mechanistic continuum. However, this hypothesis addresses what occurs after atherogenic particles cross the endothelium; it does not address the state of the endothelial barrier prior to that crossing. The second alternative — maintaining the separation — preserves a clinically distinct window. UACR, the proposed biomarker for F3, predicts cardiovascular events independently of LDL-C across diabetic and non-diabetic populations (39), and its pathogenesis in insulin-resistant states involves podocyte and endothelial injury through mechanisms that do not require lipoprotein retention (42). Furthermore, SGLT2 inhibitors and GLP-1 receptor agonists reduce both UACR and cardiovascular events, suggesting that the endothelial and barrier processes captured by F3 are therapeutically modifiable (40, 41).

Recent evidence from five independent cohorts strengthens this position. In the NEFRONA study (*n* = 1,548 Spanish patients with chronic kidney disease (CKD) and diabetes, 24-month follow-up), the combination of CKD and diabetes produced subclinical plaque in 81.4% of patients vs. 64.1% in CKD alone, with median UACR of 138 vs. 83 mg/g — diabetes effectively abolished the predictive value of traditional risk factors such as smoking and dialysis, while women with diabetes reached the same subclinical atherosclerosis burden as men without diabetes (43). In MESA (*n* = 5,581 multi-ethnic participants), longitudinal UACR trajectories independently predicted incident heart failure (HR 2.45), heart failure with preserved ejection fraction (HR 3.28), and atrial fibrillation, with 30%–40% of high-trajectory patients having baseline UACR below 30 mg/g — the conventional albuminuria threshold (44). In the German NAKO cohort (*n* = 7,613), very-low-grade albuminuria (UACR <30 mg/g) was independently associated with arterial stiffness measured by brachial-ankle PWV even in participants without hypertension, diabetes, or kidney disease (45). In a Shanghai cohort of 1,119 patients with type 2 diabetes without established cardiovascular or kidney disease, subclinical albuminuria within the conventionally normal range (UACR 6–30 mg/g) was associated with early arterial stiffness, with a threshold effect at 12.6 mg/g in postmenopausal women (46). Finally, in a three-month clinical intervention study of patients with obstructive sleep apnea and metabolic syndrome, positive airway pressure therapy reduced UACR from 86 to 17 mg/g, with visceral adiposity and HOMA-IR as independent predictors of the magnitude of UACR reduction (47). Together, these cohorts establish that albuminuria — including its sub-clinical range — captures a vascular injury signal that is prognostically independent of established lipid burden, that is modifiable through metabolic and respiratory interventions, and that emerges before the biomarkers of organized retention become abnormal.

CASCADE adopts the second alternative while explicitly acknowledging the uncertainty. The temporal relationship between barrier dysfunction and lipoprotein retention remains incompletely established in human atherosclerosis, and these processes likely overlap in many patients. The separation is proposed as a testable hypothesis: if UACR and apoB fail to identify distinct risk phenotypes in prospective cohorts, fusion into a single phase would be warranted.

F3 represents a transition zone — the last stage where the cascade may be halted before organized plaque develops, even as individual patients may simultaneously exhibit early features of organized retention. Recent intervention data illustrate this clinical complexity: in patients with type 2 diabetes, low-dose colchicine reduced mean arterial pressure (MAP)-adjusted central PWV and high-sensitivity CRP without modifying urinary albumin-to-creatinine ratio (48), consistent with the hypothesis that interventions effective at the inflammatory and vascular-stiffening levels do not necessarily reverse barrier dysfunction at the same rate.

### F4: atherogenic lipoprotein retention

F4 represents the core of classical atherosclerosis theory, and CASCADE does not seek to diminish its centrality. Once atherogenic particles — primarily those containing apoB — cross a dysfunctional endothelium, they are retained in the subendothelial space through electrostatic interactions with extracellular matrix proteoglycans. Retained lipoproteins undergo oxidative modification, triggering monocyte recruitment via CCL2 and neutrophil recruitment via CXCL1, with circadian rhythmicity adding a temporal dimension seldom considered in clinical risk assessment (9, 49). What CASCADE adds to this well-established mechanism is temporal context: in the proposed framework, retention occurs within a vascular bed that has already been conditioned by F1 through F3.

apoB is proposed as the dominant biomarker for this phase, reflecting the total burden of atherogenic particles more accurately than LDL-C alone — a position supported by current ESC and ACC/AHA guidelines, which recognize apoB as a superior metric in cases of lipid discordance (17, 50). The cumulative dimension of this process is clinically decisive. In the CARDIA cohort, 25-year LDL-C area-under-the-curve predicted cardiovascular events even in individuals with a coronary artery calcium score of zero (HR 3.36, 95% CI: 1.41–8.04 for the highest vs. lowest quartile), with each 100 mg/dL-year increment conferring 6% additional risk (51). This finding operationalizes the broader cumulative-exposure model articulated by Ference, Braunwald and Catapano, in which each individual is proposed to carry a personal threshold for plaque burden, determined jointly by lifetime atherogenic particle exposure and concurrent arterial-wall injury from hypertension, diabetes, and smoking (52). The CARDIA observation that events occur even when coronary artery calcium score (CAC) remains zero is consistent with this model: the threshold can be crossed before calcification becomes detectable.

Direct interventional evidence supports the clinical relevance of acting at this phase. The VESALIUS-CV trial randomized 12,257 patients with atherosclerosis or diabetes but without prior myocardial infarction or stroke to evolocumab or placebo; over 4.6 years, evolocumab reduced major adverse cardiovascular events by 25% (HR 0.75, 95% CI: 0.65–0.86), with on-treatment LDL-C reaching approximately 45 mg/dL (53). A prespecified subanalysis in patients with diabetes but no established atherosclerosis showed a 31% reduction (HR 0.69, *p* = 0.009) (54). Together with the cumulative-exposure findings of CARDIA, these results validate CASCADE's central premise: intervention before clinical events — and specifically at the phase of atherogenic retention — meaningfully alters outcomes.

How far this reasoning extends downward has now been tested directly. In an open-label randomized trial of 3,048 patients with atherosclerotic cardiovascular disease, targeting LDL-C below 55 mg/dL rather than below 70 mg/dL reduced the primary composite outcome over three years (HR 0.67, 95% CI: 0.52–0.86), with neutral safety across new-onset diabetes, cancer, and haemorrhagic stroke (55). The residual concerns raised about very low concentrations — a possible excess of haemorrhagic stroke, dose-dependent new-onset diabetes with statins that has not been observed with newer lipid-lowering agents, and inconsistent data on cataract — have been reviewed without evidence of a cancer or neurodegenerative signal (56). For CASCADE, this reinforces the position of F4 as the phase at which lipid burden remains modifiable; how far apoB should be driven is a question of therapeutic titration, not of phase assignment.

F4 marks the transition from the largely reversible phases (F0–F2), through the F3 transition zone, to phases in which structural change is no longer undone by treatment. The distinction is architectural rather than quantitative: aggressive LDL-lowering can reduce plaque lipid content and reinforce fibrous cap collagen on intravascular imaging, but coronary calcification represents the calcific healing of disrupted plaque and reflects accumulated structural remodeling that lipid normalization does not undo (52). What is lost across the F3–F4 transition is therefore not lipid burden — which remains modifiable — but the original arterial architecture itself.

### F5: plaque vulnerability and biological heterogeneity

Once organized plaque exists, what determines clinical outcomes is not its mere presence but its biology. F5 captures the heterogeneous processes that make plaques dangerous: inflammation within the lesion, necrotic core expansion, thin fibrous cap formation, microcalcification, and plaque erosion. These are not simply an advanced version of F4; they represent a qualitatively distinct biological state with its own drivers and biomarkers.

Lp(a) is proposed as the dominant biomarker for this phase. Genetically determined and not meaningfully modified by current lipid-lowering therapies, Lp(a) generates plaque with characteristics distinct from those driven by LDL-C alone: more inflammatory, richer in oxidized lipids, and more prone to instability (57). The PROSPECT II substudy quantified this distinction: while LDL-C, non-high-density lipoprotein cholesterol (non-HDL-C), and total cholesterol were associated with diffuse pancoronary lipid deposition and overall plaque burden, elevated Lp(a) was specifically associated with the presence of focal high-risk vulnerable plaques [*P* = 0.01 for Lp(a) alone] (58). An international consensus — the Brussels Declaration on Lp(a) testing — has called for universal Lp(a) measurement, reinforcing the clinical rationale for including this biomarker in routine cardiovascular assessment (59). The emerging therapies targeting Lp(a) directly — olpasiran, lepodisiran, and muvalaplin — represent the therapeutic frontier of this phase. Actionable strategies for patients with high Lp(a) have been recently reviewed, emphasizing that while no approved Lp(a)-specific therapy exists, aggressive management of coexisting risk factors and monitoring of subclinical atherosclerosis remain essential (60).

Vascular smooth muscle cell plasticity — encompassing clonal expansion, transdifferentiation toward proinflammatory phenotypes via KLF4, and stabilizing phenotypes via TCF21/Notch — is now recognized as a central determinant of plaque fate (61). Systemic inflammatory tone modulates this fate beyond local processes alone: in the Rotterdam Study, Mendelian randomization (MR) analysis using genetic instruments for hs-CRP supported a causal effect on PWV, with no concordant evidence for a causal role of LDL-C or HDL-C in arterial stiffness (62). This finding situates inflammation as an upstream contributor to vascular biology that intersects with the local plaque-level processes described here. Plaque vulnerability thus emerges not only from inflammatory degradation of the extracellular matrix but also from bioenergetic collapse within stabilizing smooth muscle cells, where oxidized LDL triggers miR-125a-mediated suppression of tafazzin, causing mitochondrial dysfunction and apoptosis (63). Sphingolipid metabolism provides a molecular thread across multiple phases: sphingomyelin promotes subendothelial LDL retention and aggregation (F3–F4), ceramides accumulate in vulnerable plaque near the necrotic core (F5), and sphingosine-1-phosphate exerts dual vasoprotective and pro-atherogenic effects depending on receptor subtype (F2) (64).

The intracellular cascade described above requires the cellular uptake of oxidized lipoproteins into endothelial cells and intimal macrophages, a step largely mediated by the lectin-like oxidized LDL receptor-1 (LOX-1). LOX-1 is positioned mechanistically downstream of subendothelial retention rather than as a primary driver of trapping: lipoprotein particles must first be retained within the matrix and locally modified before becoming substrates for LOX-1 internalization, a sequence consistent with the retention-first paradigm articulated for atherogenic lipoprotein retention (65). Cross-sectional clinical data support this positioning: in 968 participants from the BioHEART-CT cohort undergoing coronary computed tomography angiography (CCTA), soluble LOX-1 was independently associated with plaque burden scored by Gensini and soft-plaque indices but not with coronary artery calcium, identifying the receptor more closely with vulnerable plaque biology than with calcified disease (66). Prospective evidence in primary prevention is concordant: in 4,658 individuals from the Malmö Diet and Cancer cohort followed for 19.5 years, those in the highest tertile of plasma soluble LOX-1 had a hazard ratio of 1.76 (95% CI 1.40–2.21) for first-time myocardial infarction compared to the lowest tertile, with the association persisting after adjustment for conventional risk factors (67). Clinically, however, pharmacologic targeting of the receptor has yet to translate into measurable benefit on plaque imaging endpoints: in the GOLDILOX-TIMI 69 randomized trial of 423 post-myocardial-infarction patients with residual inflammation, the LOX-1 antagonist antibody MEDI6570 reduced soluble LOX-1 and interleukin-6 (IL-6) levels at all three doses but did not significantly reduce non-calcified plaque volume in the most diseased coronary segment over nine months (68). The negative trial does not refute the mechanistic role of LOX-1 in plaque biology so much as it underscores the redundancy of scavenger-receptor pathways in established disease and the difficulty of reversing structural plaque burden through single-receptor blockade at this stage of the cascade.

Inflammation within the plaque follows its own logic. Resolution of inflammation, mediated by specialized pro-resolving mediators (SPMs), is defective in advanced lesions compared to early lesions, creating a self-perpetuating inflammatory loop (69). This concept of “failed resolution” suggests that F4–F5 are not merely progressive stages but that F5 involves a qualitative shift where the plaque's internal biology becomes self-amplifying. The anti-inflammatory trials — CANTOS (canakinumab targeting interleukin-1β [IL-1β]) (70), COLCOT (colchicine after acute coronary syndrome [ACS]) (71), and LoDoCo2 (colchicine in chronic coronary artery disease [CAD]) (72) — provide clinical proof that inflammation is a modifiable component of plaque vulnerability, independent of lipid lowering. This interplay between inflammation and other F5 drivers extends to Lp(a). In a primary prevention analysis of 34,092 UK Biobank participants without cardiovascular disease at baseline, elevated Lp(a) was associated with increased cardiovascular events primarily among individuals with elevated interleukin-6, with the association attenuating among those with lower IL-6 levels (73). The authors caution that the signal was male-driven, attenuated at the clinically used threshold of ≥125 nmol/L, and based on IL-6 measurements on a relative platform scale rather than against validated clinical cut-offs — limitations that constrain immediate clinical application but reinforce the conceptual point that inflammatory tone modulates the expression of structural F5 biology. Residual cardiovascular risk beyond LDL-C — encompassing inflammation, remnant cholesterol, and Lp(a) — is a clinical challenge requiring targeted strategies (74).

A recent international Delphi consensus involving 35 experts proposed a change in how subclinical atherosclerosis should inform treatment: any coronary plaque detected by CT angiography warrants standard lipid-lowering therapy, and plaque volume above the age- and sex-adjusted 70th percentile warrants high-intensity treatment (75). Low-attenuation plaque — a marker of lipid-rich, vulnerable lesions — was the strongest predictor of acute myocardial infarction (HR 1.60). Notably, 25% of major adverse cardiovascular events in their analysis occurred in patients who previously had CAC scores of zero.

Real-world evidence reinforces the structural nature of this residual risk: in 20,490 patients with coronary heart disease in Stockholm followed for a mean of 2.6 years, MACE rates were comparable between low-moderate and high-intensity lipid-lowering therapy among those with good adherence (≥80%), despite the expected differences in LDL-C goal attainment between treatment intensities (76). The persistence of risk despite optimized LDL-C lowering is consistent with the framework's assignment of F5 biology to processes — plaque inflammation, fibrous cap vulnerability, and Lp(a)-driven heterogeneity — that are not adequately addressed by lipid-targeting therapies alone.

F5 is not reversible at the level of its genetic determinant, and with current therapy the objective shifts from reversal to stabilization.

### F6: subclinical atherosclerosis

When atherosclerosis becomes detectable by imaging, the patient transitions from theoretical risk to documented disease. CAC — measured by non-contrast CT — is proposed as the dominant biomarker for this phase, consistent with its established role in reclassifying treatment intensity in current guidelines (17, 50).

The population-level relevance of this phase is illustrated by the SCAPIS cohort: among 28,307 Swedish adults aged 50–64 without known cardiovascular disease, a latent-class analysis of this cohort showed the high-risk metabolic class reaching a mean predicted CAC of 92.1 compared to 42.6–43.0 in normolipidemic classes (77). A CCTA atlas of 16,300 patients across 42 Chinese centers demonstrated a 20-year delay in atherosclerosis progression in women compared to men, with plaque clustering proximal to bifurcations — consistent with hemodynamic predisposition at F3 influencing F6 distribution (78). Population-scale data now document how early this phase begins: in the REACT cohort of 16,808 adults aged 18–70 years in Denmark and Spain, assessed by three-dimensional vascular ultrasonography and coronary CT angiography, silent atherosclerosis was present in 57% of participants overall and already in 8.7% of men and 6.7% of women aged 18–29 years; with increasing age, plaque volume rose exponentially and multiarterial involvement became more frequent, whereas isolated coronary-artery atherosclerosis was uncommon in every age stratum (79).

However, CASCADE positions CAC as an adjunct to the preceding phases, not as a replacement for upstream evaluation. This aligns with Zheutlin and Greenland's editorial argument that risk equations should precede CAC testing, that a CAC=0 does not exclude noncalcified plaque (present in >10% of patients in SCOT-HEART), and that in specific populations — patients with diabetes (8.9 events per 1,000 person-years with CAC=0) and individuals with familial hypercholesterolemia (45% with CAC=0) — calcium-based reassurance can be misleading (80). The philosophical alignment with CASCADE is direct: the pressure layer (F0) and phases F1–F5 must be evaluated before F6, because a normal CAC does not mean a normal cascade.

F6 is not reversible in its structural component: established coronary calcium does not regress, and plaque burden can be stabilized but not removed. The clinical objective is reclassification of treatment intensity.

### F7: clinical cardiovascular disease

The final phase represents the clinical manifestation of the cumulative burden: myocardial infarction, stroke, peripheral artery disease, or other atherosclerotic events. By definition, every patient in F7 has traversed some combination of the preceding phases, though the path may not have been linear.

This phase is not an endpoint, but a transition to a different clinical paradigm in which the management differs from earlier phases. Secondary prevention becomes the dominant framework, organized around four therapeutic intervention targets that CASCADE helps to articulate.

First, antithrombotic and antiplatelet strategies operate as long-horizon decisions whose consequences extend over decades. The 10-year follow-up of the HOST-EXAM trial demonstrated that the choice of antiplatelet regimen after coronary stenting carries measurable cumulative consequences across a decade-long observational window (81). This illustrates a broader principle: in F7, every therapeutic decision compounds.

Second, biomarkers of cardiac stress and subclinical myocardial injury — N-terminal pro-B-type natriuretic peptide (NT-proBNP) and high-sensitivity cardiac troponin T (hs-cTnT) — provide ongoing surveillance information that complements the upstream phase-specific markers. They do not replace earlier biomarkers; they extend the monitoring framework into the post-event landscape.

Third, residual risk extends beyond lipid-centered targets along the same architectural lines that organize the upstream cascade. Persistent environmental and behavioral exposures — air pollution, suboptimal dietary patterns, insufficient physical activity, and obstructive sleep apnea — continue to activate F0 after the index event, with documented effects on event recurrence (11, 19, 47). Chronic psychosocial stress and depression operate through neuroendocrine and inflammatory pathways that parallel the F0 environmental axis. Sarcopenia and persistent visceral adiposity reflect the continued activation of F1 metabolic dysfunction in the post-event setting, contributing to functional decline and mortality independently of the index lesion. Coagulation and metabolic-vascular pathways not captured by apoB or hs-CRP — including fibrinogen and uric acid — provide additional prognostic information at the F4–F5 level. And medication adherence, the operational determinant most consistently associated with event recurrence, modulates the impact of all of the above. A fixed-dose combination strategy reduced recurrent events after myocardial infarction (82), and registry-based prevention programmes describe how lipid-lowering management can be made sustainable in routine practice (83). CASCADE positions these factors as F7 modifiers grouped by the upstream phase they reactivate, providing a structured way to identify and address them in routine secondary prevention.

Fourth, vascular age emerges as the integrative concept that ties F7 back to the entire preceding cascade. The discrepancy between chronological and vascular age — the concept of vascular resilience (84) — captures the cumulative biological trajectory across all preceding phases. A patient who reaches F7 at age 45 has a different biological trajectory than one who arrives at 75, and the speed of arrival is itself prognostic information.

Beyond these four targets, management in F7 is also shaped by risks that are transient rather than cumulative, for which CASCADE offers no phase criterion. In patients with atherosclerotic cardiovascular disease who also have atrial fibrillation, whether oral anticoagulation is continued after apparently successful catheter ablation is governed by thromboembolic risk rather than by atherosclerotic phase: in a meta-analysis of 267,443 patients, discontinuation in selected patients was associated with fewer major bleeding events without a significant increase in thromboembolism, in populations not stratified by coronary disease (85). Likewise, a transient risk of malignant ventricular arrhythmia — after a recent acute coronary syndrome, with newly diagnosed severe ventricular dysfunction, in myocarditis, or in other potentially reversible conditions — may warrant a wearable cardioverter-defibrillator as temporary protection while that risk is reassessed: appropriate interventions occurred in approximately 3% of users across 40 studies, concentrated in the first 60 days (86), and multiparametric evaluation during the wearing period may help avoid unnecessary defibrillator implantation, although the supporting series are small (87). These conditions are not phases of atherosclerosis; they are modifiers of how F7 is managed once a patient has reached it.

F7 cannot be undone at the level of past events, but the therapeutic objective remains active: event prevention, residual risk reduction, and functional preservation across the years that follow.

### Reversibility gradient

Across all phases, CASCADE proposes a reversibility gradient with direct clinical implications. F0–F2 (green zone) are largely reversible through behavioral and pharmacological intervention. F3 represents a transition zone — the last window where the cascade may be halted before organized plaque develops. F4–F7 (red zone) involve structural changes that can be stabilized but not reversed.

The distinction between zones is architectural, not quantitative. Aggressive LDL-lowering reduces plaque lipid content and reinforces fibrous cap collagen on intravascular imaging, but coronary calcification reflects healed disrupted plaque (52). What may be lost across the proposed F3–F4 transition is not modifiable lipid burden but the original arterial architecture: medial elastin organization, structural calcification, and fibrous cap geometry. These elements can be protected from further degradation but they do not regenerate.

This gradient suggests that the clinical yield of comprehensive assessment is highest in the early phases — precisely where conventional risk scores provide the least information.

## Section 2: vascular aging as a cross-cutting dimension

A defining feature of CASCADE is the treatment of vascular aging not as an additional phase but as a biological dimension that traverses and modulates every stage of the atherosclerotic cascade. This architectural decision reflects a fundamental insight: arteries do not merely accumulate plaque — they age, and the rate at which they age determines both the speed and the clinical expression of atherogenesis.

### From theoretical phase 8 to cross-cutting dimension

The initial design of CASCADE included vascular aging as F8 — a terminal evaluation of how much the vasculature had aged over the course of the disease. This approach was abandoned after considering the evidence that vascular aging begins in childhood, operates in parallel with every phase of the cascade, and is not merely an outcome of atherosclerosis but a modulator of its trajectory. The ESC 2025 cardiovascular aging review formalized this understanding, documenting the molecular mechanisms — telomere attrition, cellular senescence and the senescence-associated secretory phenotype (SASP), epigenetic drift, mitochondrial dysfunction, and clonal hematopoiesis of indeterminate potential (CHIP) — that link vascular aging to atherosclerotic progression at every phase (14). Approximately 10% of individuals over 65 harbor CHIP mutations, which accelerate both biological aging and atherogenesis through a shared inflammatory pathway.

Among these mechanisms, CHIP provides perhaps the most direct causal link between aging and atherogenesis. Somatic mutations in hematopoietic stem cells — most commonly in TET2, DNMT3A, and ASXL1 — expand clonally with age and give rise to macrophages with a hyperinflammatory phenotype that accelerates plaque formation and instability. In prospective cohorts, CHIP carriers have an approximately twofold increased risk of coronary heart disease (hazard ratio 1.9, 95% CI 1.4-2.7), and TET2 loss-of-function in murine models directly accelerates atherosclerosis through enhanced NLRP3 inflammasome activation (88).

A parallel body of work has elucidated the systemic epigenetic architecture of aging. In a comprehensive analysis of 22 cell types, AP-1 was identified as a pioneer transcription factor that hijacks cell-identity enhancers toward a SASP/inflammatory program during aging, while FOXM1 — a cyclically expressed transcription factor — extends lifespan by 29% when overexpressed, acting through AP-1 suppression. EZH2, a component of the Polycomb Repressive Complex 2 (PRC2), emerged as the most effective transcription factor for cellular rejuvenation in a systematic screen. The PRC2-AgeIndex, derived from these findings, has been validated as a biomarker of rejuvenation by partial reprogramming *in vivo* (13). Notably, Klotho — a key renoprotective and anti-aging factor — is epigenetically silenced by PRC2 during renal aging, providing a molecular bridge between vascular aging and the endothelial/renal processes captured by F3.

### Phenotypes: early vascular aging (EVA), normal aging, and super-normal vascular aging (SUPERNOVA)

The discrepancy between chronological and vascular age defines three phenotypes with distinct cardiovascular trajectories. Early Vascular Aging (EVA) describes individuals whose arterial stiffness exceeds what is expected for their age — a condition that can be detected from childhood and adolescence according to the Youth Vascular Consortium expert consensus (89). Normal vascular aging follows the expected trajectory. Super-Normal Vascular Aging (SUPERNOVA) describes individuals with arterial stiffness below age-expected values, conferring significant protection: in the Malmö Diet and Cancer cohort (*n* = 2,642; 6.6 years of follow-up), SUPERNOVA individuals had a hazard ratio of 0.59 (95% CI: 0.41–0.85) for cardiovascular events (90).

These phenotypes are not merely descriptive labels. They suggest that the velocity of transit through CASCADE's phases varies between individuals in ways that chronological age alone cannot capture. A 45-year-old patient with EVA may be biologically traversing F4 at the speed of a 65-year-old, while a 70-year-old with SUPERNOVA may retain F2 vascular characteristics. This has direct clinical implications: the intensity and urgency of intervention at any given phase should be modulated by the patient's vascular aging phenotype.

### Biological clocks

Multiple biological aging clocks are now available, each capturing different dimensions of the aging process. Their relevance to CASCADE lies in the fact that many of their components overlap with the biomarkers assigned to specific phases, creating a natural convergence between aging measurement and atherosclerosis staging.

Epigenetic clocks represent the most mature category. First-generation clocks (Horvath, Hannum) estimate chronological age from DNA methylation patterns. Second-generation clocks incorporate clinical outcomes: PhenoAge integrates albumin, creatinine, glucose, CRP, and lymphocyte percentage — variables that map to F1, F3, and F5 in CASCADE. GrimAge is another second-generation clock. Epigenetic age acceleration has been linked to age-related diseases, mortality, and subclinical atherosclerosis (14).

Proteomic clocks offer a complementary dimension. ProtAge, derived from 204 proteins and correlating 0.94 with chronological age in UK Biobank, predicts 18 chronic diseases, multimorbidity, and mortality (91).

The inflammatory clock iAge, whose strongest contributor is the chemokine CXCL9 (92), captures the “inflammaging” component of vascular aging and maps naturally to the inflammatory processes of F5. Clinical vascular clocks based on routine laboratory values offer an accessible alternative that can be computed from data already collected in standard clinical practice. A periorbital skin age index derived from facial image analysis has been shown to correlate with chronological age (r > 0.7) and with five of seven chronic diseases tested, illustrating the search for accessible, non-invasive biological aging metrics (93).

The inflammaging component of vascular aging is empirically traceable across phases. The Rotterdam Study mediation analysis with sex-specific stratification (hs-CRP mediating age-related vascular dysfunction more strongly in men than in women, with MR support for hs-CRP→PWV causality) and the Lp(a) × IL-6 interaction observed in 34,092 UK Biobank primary-prevention participants are interpretable within this dimension as the empirical intersection of horizontal phase-progression with cross-cutting inflammatory aging. The generalizability of the eating-window data anchoring F0 is similarly constrained by the systematically healthier composition of volunteer-derived cohorts (see Limitations).

Arterial-specific measurements complete the toolkit. Carotid-femoral PWV (cfPWV) remains the gold standard for measuring arterial stiffness, with a secondary analysis of the SPRINT trial showing that estimated PWV (ePWV) predicts the primary cardiovascular composite outcome (HR 1.30, 95% CI: 1.17–1.43) and all-cause mortality (HR 1.65, 95% CI: 1.46–1.86) (94). A cfPWV threshold of >10 m/s indicates target organ damage (95), and each 1 m/s increment is associated with a 14% increase in cardiovascular event risk (96).

No single unified metric for vascular age currently exists. CASCADE acknowledges this limitation while proposing that the convergence of these clocks — each applicable at specific phases — provides operationally useful information even in the absence of a master metric.

### Convergence: where vascular aging meets the cascade

CASCADE's contribution to vascular aging is the mapping of specific clocks and aging biomarkers to the phases where they have the greatest diagnostic expression. At F0 environmental exposures accelerate arterial stiffening; EVA phenotypes can be identified even in youth. At F1, clinical biomarkers of adaptive metabolic response are simultaneously components of PhenoAge and clinical aging clocks. F2 represents the first phase where vascular aging is directly measurable through PWV. At F3, endothelial injury and barrier dysfunction is a hallmark of vascular aging. F4–F5 bring maximal overlap between inflammaging markers and atherogenic processes. F6 represents peak convergence: elevated CAC, increased cfPWV, and epigenetic age acceleration by GrimAge present an integrated phenotype. At F7, the cumulative expression of vascular aging determines whether a patient reached clinical disease at 45 or at 75.

### Pharmacological modifiability

The clinical relevance of vascular aging as a dimension depends on whether it is modifiable. Emerging evidence suggests it is. In a nonhuman primate model, 40 months of metformin administration reduced proteomic age by 6.4 years, frontal lobe methylation age by 6.1 years, and organ-specific aging in kidney (−4.9 years) and liver (−3.95 years) (97). Over 20 clinical trials of metformin as a geroprotective agent are currently registered. In murine models, spermidine supplementation restored acetylhypusination of RIPK1, preventing endothelial necroptosis and the downstream cascade of microvascular damage leading to insulin resistance (31). Senolytic strategies have been shown to reduce arterial stiffness and improve endothelial function in aged preclinical models (98).

The heritability of intrinsic aging has been estimated at 55% when extrinsic mortality is properly accounted for, suggesting that genetic determinants of aging velocity are substantially larger than previously recognized (99). This finding supports the proposition that predisposition toward specific cardiometabolic trajectories may be identifiable far earlier than current practice assumes.

Type 2 diabetes itself has been described as a model of accelerated biological aging, with early-onset diagnosis (before age 40) conferring 3–4 years of life expectancy reduction (29) — reinforcing the proposition that cardiometabolic trajectories and vascular aging velocity are intertwined from early life.

Artificial intelligence approaches are beginning to model aging trajectories at single-cell resolution. In a preprint, a temporal transformer model trained on approximately one trillion gene tokens across the human lifespan predicted cell-state transitions between ages and inferred disease-associated age acceleration — including +5 years for smoking and +15 years for pulmonary fibrosis. Notably, perturbations predicted as pro-aging were validated *in vivo* with significant functional decline, and one identified driver (P4HA1) had been previously reported as downregulated by GLP-1 receptor agonists, suggesting a molecular link between geroprotective pharmacology and artificial intelligence (AI)-predicted aging mechanisms (100).

These findings support the position that vascular aging, while not yet a standard therapeutic target, is approaching clinical actionability — and that a framework like CASCADE, which maps aging to each phase of atherosclerotic disease, could help identify which patients would benefit most from geroprotective interventions.

## Section 3: proposed biomarker framework

A chronological framework without measurable anchors at each stage would be descriptive but not clinically actionable. CASCADE proposes a dominant biomarker for each phase — the single measurement that best captures the pathophysiological essence of that stage with the strongest available evidence (Table 1; the full variable set, with measurement methods and resource levels, appears in Table 2). These are proposed candidate biomarkers, not a validated risk score. Each individual marker is well-established in the cardiovascular literature; their combination as a phase-specific set is a CASCADE proposal that requires empirical validation.

**Table 1:** Dominant biomarker, reversibility zone, complementary/fallback markers, and rationale for each CASCADE phase. Biomarker selections represent expert-informed choices guided by pathophysiological representativeness, clinical accessibility, and pragmatic discrimination; they were not derived statistically from a validation cohort. Of the eight proposed dominant biomarkers, the UPF-score (F0) and the integrated vascular age metric (F7) lack external validation; the remaining six are individually well-established but have not been tested as a combined set (see Limitations). F0 is an environmental and behavioral pressure layer rather than a biological phase; the seven biological phases are F1–F7. The reversibility zone (green/yellow/red) is a conceptual gradient described in the text and rendered in Figure 1; in F2, the functional component is reversible whereas the structural component (elastin fragmentation) is largely irreversible. F5 [Lp(a)] has no fallback marker because it is genetically determined and measured once per lifetime, with no validated proxy. In F7, medication adherence and physical status are operational determinants/modifiers grouped under this phase rather than proxies of the integrative marker. Complement denotes a marker adding information of a different nature or higher resolution; fallback denotes a more accessible substitute when the dominant marker is unavailable. ACC/AHA, American College of Cardiology/American Heart Association; apoB, apolipoprotein B; CAC, coronary artery calcium; CCTA, coronary computed tomography angiography; cfPWV, carotid-femoral pulse wave velocity; CT, computed tomography; eGFR, estimated glomerular filtration rate; ESC, European Society of Cardiology; HDL-C, high-density lipoprotein cholesterol; HOMA-IR, Homeostatic Model Assessment of insulin resistance; hs-CRP, high-sensitivity C-reactive protein; hs-cTnT, high-sensitivity cardiac troponin T; LDL-C, low-density lipoprotein cholesterol; Lp(a), lipoprotein(a); LVEF, left ventricular ejection fraction; ML-AAC24, machine-learning-derived abdominal aortic calcification score (24-point scale); MR, Mendelian randomization; non-HDL-C, non-high-density lipoprotein cholesterol; NT-proBNP, N-terminal pro-B-type natriuretic peptide; PM2.5, fine particulate matter; PWV, pulse wave velocity; TyG, triglyceride-glucose index.

Phase	Phase name	Dominant biomarker	Reversibility zone	Complement/fallback	Key evidence/rationale
F0 *pressure layer*	Environmental & behavioral pressure layer *(not a biological phase)*	UPF-score *(composite ultra-processed food score)*	Largely reversible (green zone)	Alternative markers: tobacco (pack-years), PM2.5	Operational proposal; requires external validation. Likely to require a multidimensional composite (nutritional + environmental + psychosocial + physical activity) (5, 11).
F1	Adaptive metabolic response	HOMA-IR	Largely reversible (green zone)	Complement: TyG. Fallback: waist circumference.	Selected over TyG (more triglyceride-influenced; overlaps with F4). Non-linear relationship with macro-/microvascular damage; saturation threshold ∼HOMA-IR = 5; stronger in those <50 years (27).
F2	Vascular response & arterial stiffening	ePWV *(estimated pulse wave velocity)*	Largely reversible (green zone) — functional component reversible; structural component (elastin fragmentation) largely irreversible	Complement: cfPWV (measured PWV). Fallback: pulse pressure.	Derivable from age and mean blood pressure at zero cost; an accessible surrogate for cfPWV (Reference Values for Arterial Stiffness Collaboration equation, 2010); validated prognostically in an independent population-based cohort (35).
F3	Endothelial injury & barrier dysfunction	UACR *(urine albumin-to-creatinine ratio)*	Transition (yellow zone)	Complement: eGFR. Fallback: urine dipstick (proteinuria).	Most accessible systemic proxy of endothelial integrity; an indirect marker (does not measure glycocalyx directly). Predicts cardiovascular events independently of LDL-C (39, 42).
F4	Atherogenic lipoprotein retention	apoB	Stabilization only (red zone) — apoB/lipid burden still modifiable	Complement: remnant cholesterol. Fallback: non-HDL-C.	Greatest consensus; reflects total atherogenic-particle burden more accurately than LDL-C alone. ESC 2025 (50) + ACC/AHA 2026 Class IIa for apoB measurement (17). HDL-C is not proposed (U-shaped relationship; no robust MR causality).
F5	Plaque vulnerability & biological heterogeneity	Lp(a)	Stabilization only (red zone)	Complement: hs-CRP. Fallback: — (no valid proxy; see note)	Risk intensifier; genetically determined; plaque more inflammatory/unstable. The Lp(a) + hs-CRP pair is validated (105, 106).
F6	Subclinical atherosclerosis	CAC *(Agatston, non-contrast CT)*	Stabilization only (red zone)	Complement: ML-AAC24 (abdominal aortic calcification), CCTA (non-calcified plaque). Fallback: carotid ultrasound.	Established role. ACC/AHA 2026 formalizes a 4-tier ladder (1–99/100–299/300–999/ ≥ 1000); an adjunct, not a replacement for earlier phases (17, 80).
F7	Clinical cardiovascular disease	Chronological vs. vascular age *(integrated metric — within a 4-objective secondary-prevention architecture)*	Stabilization only (red zone)	Complement (extended surveillance): NT-proBNP, hs-cTnT, LVEF. Fallback: medication adherence, physical status (e.g., dynapenia).	Transition to secondary prevention, not an endpoint. Four objectives: (1) antithrombotic/antiplatelet (81); (2) surveillance (NT-proBNP + hs-cTnT); (3) residual risk grouped by reactivated upstream phase; (4) vascular age as integrator (“vascular resilience”) (84).

**Table 2:** Measurement variables by CASCADE phase, with resource level and relationship to the vascular-aging dimension. This table expands Table 1 by listing, for each CASCADE phase, the proposed dominant biomarker together with its complementary and fallback markers, the measurement method, the resource level at which it can be obtained, and its relationship to the cross-cutting vascular-aging dimension. The table reports no quantitative thresholds: CASCADE proposes no validated phase-transition cut-offs, which require empirical derivation in prospective cohorts and are expected to vary by population, age, and sex (see Limitations). Where guideline-based clinical thresholds exist for individual markers as standalone tools, they are discussed in the main text with their original sources. F0 is an environmental and behavioral pressure layer rather than a biological phase; the seven biological phases are F1–F7. Role labels: dominant = proposed primary biomarker for the phase; complement = adds information of a different nature or higher resolution; fallback = a more accessible substitute when the dominant marker is unavailable. Citations for the supporting evidence appear in the main text. apoB, apolipoprotein B; BP, blood pressure; CAC, coronary artery calcium; CCTA, coronary computed tomography angiography; cfPWV, carotid-femoral pulse wave velocity; CKD-EPI, Chronic Kidney Disease Epidemiology Collaboration; CT, computed tomography; eGFR, estimated glomerular filtration rate; ePWV, estimated pulse wave velocity; EVA, early vascular aging; HDL-C, high-density lipoprotein cholesterol; HOMA-IR, Homeostatic Model Assessment of insulin resistance; hs-CRP, high-sensitivity C-reactive protein; hs-cTnT, high-sensitivity cardiac troponin T; iAge, inflammatory aging clock; IMT, intima-media thickness; LDL-C, low-density lipoprotein cholesterol; Lp(a), lipoprotein(a); LVEF, left ventricular ejection fraction; ML-AAC24, machine-learning-derived abdominal aortic calcification score (24-point scale); non-HDL-C, non-high-density lipoprotein cholesterol; NT-proBNP, N-terminal pro-B-type natriuretic peptide; PM2.5, fine particulate matter; PWV, pulse wave velocity; SUPERNOVA, super-normal vascular aging; TC, total cholesterol; TyG, triglyceride-glucose index; UACR, urine albumin-to-creatinine ratio; UPF-score, composite ultra-processed food score.

Phase	Variable (role)	Measurement method	Resource level	Vascular-aging dimension
F0 *pressure layer*	UPF-score *(dominant)* Tobacco, pack-years *(alternative)* PM2.5 *(alternative)*	Validated dietary questionnaire (composite) Structured history/autocalculation Environmental estimate/georeferencing	Primary care Primary care Specialized/external	Environmental exposures accelerate arterial stiffening; EVA phenotypes identifiable in youth.
F1	HOMA-IR *(dominant)* TyG *(complement)* Waist circumference *(fallback)*	Fasting glucose × insulin/405 Fasting triglycerides × glucose (log formula) Standardized tape measure	Specialized lab Primary care Primary care	Adaptive-metabolic biomarkers are components of PhenoAge and clinical aging clocks.
F2	ePWV *(dominant)* cfPWV — measured PWV *(complement)* Pulse pressure *(fallback)*	Derived from age + mean blood pressure Carotid-femoral tonometry Office BP (systolic−diastolic)	Primary care (derived) Specialized Primary care	First phase where vascular aging is directly measurable; defines EVA/SUPERNOVA phenotypes.
F3	UACR *(dominant)* eGFR *(complement)* Urine dipstick — proteinuria *(fallback)*	Spot urine albumin/creatinine CKD-EPI 2021 (creatinine/cystatin C) Reagent strip	Primary care Primary care Primary care	Endothelial injury and barrier dysfunction is a hallmark of vascular aging (renal-vascular axis).
F4	apoB *(dominant)* Remnant cholesterol *(complement)* Non-HDL-C *(fallback)*	Specialized-lab immunoassay Calculated (TC−LDL-C−HDL-C) Calculated (TC−HDL-C)	Specialized lab Primary care (calculated) Primary care	F4–F5 show maximal overlap between inflammaging markers and atherogenic processes.
F5	Lp(a) *(dominant)* hs-CRP *(complement)* — no fallback *(no valid proxy)*	Specialized lab (once per lifetime) High-sensitivity assay Genetically determined; not substitutable	Specialized lab Primary/specialized lab —	Inflammaging overlap (hs-CRP as iAge component) intersects Lp(a)-driven structural heterogeneity.
F6	CAC *(dominant)* ML-AAC24 *(complement)* CCTA — non-calcified plaque *(complement)* Carotid ultrasound *(fallback)*	Non-contrast CT (Agatston) Abdominal aortic calcification (ML-scored) Coronary CT angiography B-mode ultrasound (plaque/IMT)	Imaging Imaging Imaging Imaging (accessible)	Peak convergence: elevated CAC, increased cfPWV, and epigenetic age acceleration (GrimAge) present an integrated phenotype.
F7	Chronological vs. vascular age *(dominant, integrative)* NT-proBNP, hs-cTnT, LVEF *(complement — surveillance)* Medication adherence, physical status *(fallback/modifier)*	Composite (no unified metric) Laboratory + echocardiography Structured assessment; dynamometry, gait speed	Multimodal Specialized lab/imaging Primary care	Cumulative expression of vascular aging determines whether clinical disease is reached at 45 or at 75.

### Selection criteria

Three principles guided biomarker selection. First, pathophysiological representativeness: each marker should reflect the core biological process of its assigned phase. Second, clinical accessibility: markers should be obtainable in urban clinical settings across different resource levels, with established alternatives available when the primary marker is not. Third, pragmatic discrimination: where multiple candidates existed, the marker with the strongest evidence for independent cardiovascular prediction was preferred, unless accessibility constraints favored an alternative.

These selections were not determined by statistical derivation from a validation cohort. They represent expert-informed choices that are transparently acknowledged as such.

### Phase-by-phase justification

For F0, CASCADE proposes a composite ultra-processed food score (UPF-score) as an initial operational marker. However, evidence that exposomic burden and psychosocial stress independently and substantially drive biological aging and cardiovascular risk suggests that F0 assessment will likely require a multidimensional composite integrating nutritional, environmental, psychosocial, and physical activity domains. Tobacco exposure in pack-years and PM2.5 estimates have stronger epidemiological support as individual markers and serve as established alternatives. The development and validation of such a composite represents a priority for future iterations of the framework.

For F1, HOMA-IR was selected over the triglyceride-glucose index (TyG). Both quantify insulin resistance, but HOMA-IR more directly captures the metabolic signaling pathway that CASCADE positions as the initiating adaptive response. TyG is more heavily influenced by triglyceride metabolism — a process that overlaps with F4. Additionally, the evidence that intensive triglyceride reduction translates into proportional mortality benefit remains inconsistent — the ESSENCE-TIMI 73b trial demonstrated that olezarsen, an apolipoprotein C-III (APOC3) inhibitor, reduced triglycerides by ∼64% and apoB by ∼16% (101); however, its coronary CT angiography substudy showed no significant reduction in noncalcified plaque volume at 12 months (102), indicating that triglyceride reduction with only modest apoB lowering does not translate into atherosclerotic regression — whereas interventions targeting the metabolic substrate have demonstrated cardiovascular event reduction (33). TyG is positioned as a complementary marker (specialized laboratory). Waist circumference serves as the clinical fallback.

For F2, estimated PWV (ePWV) — derivable from age and blood pressure at zero cost — was selected as an accessible surrogate for carotid-femoral PWV (cfPWV). Pulse pressure serves as a zero-cost fallback.

For F3, UACR is proposed as the most accessible systemic proxy for endothelial barrier integrity. UACR does not directly measure glycocalyx damage — it is an indirect marker. Estimated glomerular filtration rate (eGFR) is the complementary marker.

For F4, apoB is the marker with the strongest consensus support. Non-HDL cholesterol is the operational fallback where apoB measurement is unavailable. This selection is reinforced by the 2026 ACC/AHA Multisociety Dyslipidemia Guideline, which introduced apoB measurement as a Class IIa recommendation to guide further therapeutic intensification once LDL-C and/or non-HDL-C goals are achieved (17), and by independent cost-effectiveness modeling demonstrating that apoB-guided therapy outperforms both LDL-C and non-HDL-C goals, with an incremental cost-effectiveness ratio of $30,300 per quality-adjusted life-year (QALY) and optimality in 65% of probabilistic iterations (103). Notably, HDL-cholesterol is not proposed as a Phase 4 marker, reflecting accumulating evidence that HDL-C follows a U-shaped rather than a linear relationship with cardiovascular events (replicated in the Copenhagen, ChinaHEART, NHANES, and UK Biobank cohorts), that Mendelian randomization studies have not demonstrated a robust causal effect, and that recent cholesteryl ester transfer protein (CETP)-inhibitor trials (obicetrapib) have reframed this class as apoB-lowering therapy rather than HDL-C-raising therapy (104).

For F5, Lp(a) is proposed as the risk intensifier that best captures the heterogeneous biology. hs-CRP serves as the inflammatory complement. The combined predictive value of this biomarker pair has recently been validated: in the EPIC-Norfolk prospective cohort (*n* = 17,087, 20-year follow-up), a single measurement of LDL-C, hs-CRP, and Lp(a) independently and additively predicted MACE, with adjusted hazard ratios of 1.78, 1.55, and 1.19 respectively in the highest vs. lowest quintile, and a combined hazard ratio of 2.41 (95% CI: 1.90–3.07) when all three markers fell in the top quintile (105). These findings replicate prior observations from the Women's Health Study and extend them to men, validating the specific combination of lipid, inflammatory, and genetic lipoprotein markers that CASCADE assigns to F4–F5. Independent confirmation in a non-European population comes from a cross-sectional Chinese cohort (*n* = 2,788,206 adults, 30 provinces), in which elevated Lp(a) was independently associated with subclinical atherosclerosis at multiple vascular sites — carotid plaque, coronary artery calcium, carotid intima-media thickness, and subclinical cerebral infarcts — with a dose-dependent gradient across the number of affected sites (odds ratios of 1.16, 1.40, and 1.41 for one, two, and three sites respectively) (106). This extends the validity of Lp(a) as an F5 biomarker beyond European cohorts, despite median population concentrations being substantially lower than in Caucasians.

For F6, CAC requires no justification beyond its established role. The 2026 ACC/AHA Guideline formalized a four-tiered CAC ladder (1–99, 100–299, 300–999, ≥1000) with stratum-specific LDL-C and apoB targets, operationalizing what CASCADE proposes conceptually: that subclinical atherosclerosis is a continuum requiring graduated intervention intensity rather than a binary classification (17). Carotid plaque by ultrasound is the alternative. Automated abdominal aortic calcification scoring from routine lateral spine imaging has also emerged as a complementary marker of subclinical disease: in the UK Biobank Imaging Study (*n* = 50,923), machine-learning-derived ML-AAC24 was the second strongest predictor of ASCVD after age (HR 2.46 for high scores), with one in five participants having moderate-to-high scores (107). Prospective data from asymptomatic individuals confirm the clinical weight of subclinical disease: in a Danish general-population cohort of 10,003 adults over 40 years assessed by coronary CT angiography, obstructive subclinical coronary atherosclerosis conferred a fourfold higher hazard of myocardial infarction in women and a 12-fold higher hazard in men over a median follow-up of 6 years (108).

For F7 CASCADE proposes the discrepancy between chronological age and vascular age, complemented by NT-proBNP. The vascular age concept does not yet have a single unified metric.

### Phase assignment

Assigning an individual to a phase requires an explicit rule, and CASCADE proposes a qualitative and hierarchical one rather than a score. A patient is placed in the most advanced phase for which the corresponding dominant biomarker is abnormal; earlier phases are understood as persisting rather than as superseded, so that a patient in F4 typically still carries the metabolic and arterial abnormalities of F1 and F2. Where an established guideline threshold exists for a dominant biomarker, CASCADE adopts that threshold rather than proposing a new one; where no consensus threshold exists, the framework states this explicitly and reads the marker qualitatively. The cut-points below are therefore borrowed, not derived: they carry the evidence — and the uncertainty — of the guidelines from which they come, and they should be expected to change as those guidelines do.

For F1, no single consensus cut-point for insulin resistance is available: HOMA-IR and the triglyceride-glucose index are both in use, their distributions are population-specific, and the framework asks only for evidence of elevated insulin resistance by locally validated criteria (10, 27, 28). For F2, a carotid-femoral pulse wave velocity above 10 m/s is the established marker of hypertension-mediated organ damage (95), with ePWV as the accessible bridge where cfPWV is unavailable (35). For F3, a urine albumin-to-creatinine ratio of 30 mg/g or higher corresponds to Kidney Disease: Improving Global Outcomes (KDIGO) category A2 (39, 44–46); values between 10 and 30 mg/g are not cardiovascularly neutral and are read as an early signal within the F3 transition zone rather than as a separate category. For F4, apoB is interpreted against the risk-stratified targets of current lipid guidelines (17, 50) rather than against a CASCADE-specific number. For F5, an Lp(a) concentration of 125 nmol/L or higher (approximately 50 mg/dL) identifies the high-risk stratum at which the phase becomes informative (59, 60, 74), while lower concentrations remain relevant to overall risk assessment. For F6, any coronary artery calcium score above zero places a patient in the phase, and a score of 100 or higher — or above the 75th percentile for age and sex — supports reclassification to high risk (17, 50, 51, 80). F7 is defined by a documented clinical event and needs no threshold. Vascular aging is deliberately left without a numerical algorithm: it is read as a modifier of the pace at which a patient traverses the phases, not as a criterion for entering one.

Three qualifications follow from this rule. First, phase entry is not necessarily sequential: monogenic conditions bypass the upstream phases, so that a patient with familial hypercholesterolemia enters at F4 and a patient with genetically elevated Lp(a) at F5 without having traversed F1 through F3 — the framework describes a common trajectory, not an obligatory one. Second, the thresholds above are population-level conventions and should be expected to vary with age, sex, ancestry, diabetes, chronic kidney disease, and established cardiovascular disease; coronary artery calcium is already read against age- and sex-specific percentiles, and CASCADE anticipates that its remaining anchors will require comparable calibration. Third, a change of phase during follow-up is defined by the same rule applied to a repeat measurement, confirmed rather than assumed from a single value; how often such transitions occur, and how reproducibly a phase can be assigned by different clinicians, are questions the validation pathway outlined below is intended to answer.

### Feasibility and tiered access

Not all eight markers are equally accessible. In a primary care setting without specialized equipment, three dominant markers can be obtained directly — UPF-score (questionnaire), ePWV (derived from age and blood pressure), and UACR (urine sample) — and two further phases can be approximated through their fallbacks, waist circumference for HOMA-IR (F1) and non-HDL-C for apoB (F4), so that five of the eight phases can be staged at this level. HOMA-IR, apoB, and Lp(a) require a specialized laboratory request [Lp(a) once per lifetime], CAC requires referral to an imaging center, and the F7 integrative metric is multimodal. This tiered structure is intentional: CASCADE is designed to provide clinically useful information at multiple resource levels, expanding in resolution as additional measurements become available.

Implementation is a further challenge that positioning alone does not resolve. Several of the proposed anchors — apoB, Lp(a), coronary artery calcium scoring — remain heterogeneously available and reimbursed across health systems, and HOMA-IR requires an insulin assay that many primary-care laboratories do not run. Adopting CASCADE therefore implies training clinicians to think in phases rather than only in risk scores, and its cost-effectiveness relative to calculator-based strategies is unknown. A staged adoption path is plausible — beginning with the zero-cost reinterpretation of measurements already ordered, and adding phase-specific anchors only where they would change management — but demonstrating feasibility is part of the validation agenda, not a premise of the framework.

## Section 4: clinical and operational implications

CASCADE is not a risk calculator; instead, it provides an organizational architecture that changes how atherosclerotic risk is assessed, communicated, and acted upon — particularly in the early, reversible phases that conventional tools do not address.

### Reframing the clinical encounter

Current risk stratification begins, in practice, with a lipid panel and a 10-year risk estimate. A patient with normal LDL-C, no diabetes, and a low Framingham or SCORE2 score is typically reassured. CASCADE proposes that this assessment is incomplete. The same patient may have an ultra-processed dietary pattern (F0), elevated HOMA-IR (F1), an ePWV above age-expected values (F2), or an elevated UACR (F3) — all of which precede the lipid-centered window where conventional scores operate. By organizing these observations into a chronological sequence, CASCADE offers the clinician a structured way to identify where in the cascade a patient currently stands and which interventions are most relevant at that stage.

This is particularly consequential for primordial and early primary prevention — the clinical space where the greatest potential for disease modification exists but where current tools provide the least guidance. Framingham and SCORE2 were designed to estimate event probability in the intermediate-to-high risk window (in our interpretation, primarily F4 and F7). PREVENT expands this scope but remains a statistical estimator rather than a framework that maps where the patient is in the disease process. CASCADE complements these tools by providing upstream context (Table 3).

**Table 3:** CASCADE compared with conventional cardiovascular risk scores: phase coverage and scope. Coverage matrix: ● indicates that the tool addresses the corresponding CASCADE phase. VA = cross-cutting vascular-aging dimension. The phase mapping is an interpretation by CASCADE of the pathophysiological window each tool addresses, based on the variables each tool uses; it is not a property declared by the original developers of each score. Framingham, SCORE2, and GLOBORISK are 10-year risk estimators built on classical risk factors and map to the established-disease window (F4 and F7); capturing diabetes as a categorical antecedent is not equivalent to assessing the adaptive-metabolic phase (F1), which CASCADE anchors on insulin resistance, so F1 is left unmarked for these tools. PREVENT additionally incorporates kidney function and metabolic markers, extending partial coverage to F1 and F3, but remains a statistical estimator rather than a framework that maps where a patient stands in the disease process. GLOBORISK is mapped here from its published input variables (age, sex, blood pressure, total cholesterol, diabetes, smoking), as the main text does not describe it individually. CASCADE is an organizational architecture rather than a risk calculator and is positioned as complementary to these tools, providing chronological context across all phases. BP, blood pressure; VA, vascular aging.

Tool	Type	Key variables	F0	F1	F2	F3	F4	F5	F6	F7	VA	Principal limitation/what it leaves out
Framingham	10-year risk estimator	Age, sex, BP, cholesterol, diabetes, smoking					●			●		No upstream metabolic/vascular phases; no chronological sequence; North American cohort
SCORE2	10-year risk estimator	Age, sex, BP, lipids, smoking (European calibration)					●			●		Operates within established lipid-burden window; no early metabolic/vascular processes
GLOBORISK	10-year risk estimator (country-recalibrated)	Age, sex, BP, total cholesterol, diabetes, smoking					●			●		Same classical risk-factor window as Framingham/SCORE2; no upstream or imaging phases
PREVENT	10-year/30-year risk estimator	Classical factors + kidney function, metabolic markers, social determinants		●		●	●			●		Broader multisystem scope, but remains a statistical estimator; does not map disease-process position
CASCADE	Chronological organizational architecture (not a risk score)	Phase-specific dominant biomarkers F0–F7 + cross-cutting vascular aging	●	●	●	●	●	●	●	●	●	No outcome data of its own; phase-specific biomarker set requires prospective validation

### The reversibility gradient as communication tool

The proposed reversibility gradient — green (F0–F2), yellow (F3), red (F4–F7) — has a direct application in patient communication. Telling a patient “you are in F1, the most modifiable biological phase of the cascade” carries a different motivational weight than “your 10-year risk is 6%.” The phase-based framing makes the biological state tangible: it is not a probability of something happening in the future, but a description of something happening now.

The reversibility gradient also informs clinical urgency. A patient in F3 — with elevated UACR but no apoB elevation — may represent the last window before organized plaque develops. Conversely, a patient in F6 with high CAC has crossed into the stabilization zone and requires reclassification of lipid targets regardless of the calculated 10-year risk.

### Applicability across prevention categories

In primordial prevention (F0–F1), CASCADE identifies individuals with environmental and metabolic exposures before any vascular or structural changes are detectable. In primary prevention (F2–F6), CASCADE provides a chronological roadmap for escalating assessment and intervention. In secondary prevention (F7), CASCADE recontextualizes the post-event patient by asking not only “what happened” but “through which phases did this patient arrive here.”

### The cumulative exposure framework as kinetic anchor

The chronological organization proposed by CASCADE aligns with a broader recognition that atherosclerotic risk is determined by the cumulative interaction of magnitude, duration, and timing of exposure rather than by cross-sectional measurements alone. Ference, Braunwald and Catapano (52) formalized this view as the cumulative LDL exposure hypothesis, in which each individual carries a personal plaque threshold determined by lifetime apoB-particle burden and concurrent arterial wall injury from hypertension, diabetes, and smoking. This framing — articulated in a top-tier review by senior figures in cardiovascular prevention — provides the kinetic foundation on which a phase-based architecture becomes operationally meaningful: if risk integrates over decades, then mapping where in the trajectory a patient currently stands is not an alternative to risk estimation but a prerequisite for it.

### Positioning CASCADE within the current debate

Contemporary discussion of atherosclerosis prevention reflects three partially overlapping paradigms, each emphasizing a different driver of disease. The inflammation-centric view — grounded in the JUPITER, CANTOS, COLCOT, and LoDoCo2 trials, and reinforced by the observation that up to 25% of patients hospitalized with myocardial infarction present without any of the four classical risk factors — argues that inflammation is a modifiable determinant of vulnerability independent of lipid burden. The lipid-centric view — articulated through the response-to-retention framework and reinforced by decades of trial evidence from statins, ezetimibe, proprotein convertase subtilisin/kexin type 9 (PCSK9) inhibitors, and newer therapies — maintains that apoB-containing particle retention is the necessary and dominant cause of atheroma, and that inflammation is a downstream consequence rather than an independent driver. The imaging-centric view — supported by CCTA follow-up data, plaque-directed therapy trials, and the 2026 ACC/AHA CAC-based intensity ladder — proposes that direct visualization and serial measurement of plaque should guide preventive intensity, rather than inference from upstream surrogates. CASCADE suggests that these are not competing hypotheses but complementary perspectives operating at different phases of a chronological continuum. Vascular response, arterial stiffening, endothelial injury and barrier dysfunction (F2–F3) precede retention and have therapeutic windows during which anti-inflammatory and endothelium-preserving interventions are biologically plausible, though not yet trialed in that specific window. apoB-containing particle retention (F4) is the dominant driver once retention begins, and lipid-lowering interventions at this stage have the strongest trial evidence. Plaque visualization (F5–F6) becomes most informative once the preceding phases have produced detectable structural change. The chronological architecture reconciles the three paradigms by assigning each its biologically appropriate window rather than treating them as mutually exclusive alternatives.

CASCADE should also be read alongside existing staging and classification systems, not only alongside the risk calculators compared in Table 3. The ACC/AHA heart-failure stages A–D established the precedent that a chronic cardiovascular disease can be staged by biological progression rather than by symptoms alone; CASCADE proposes the same move for atherosclerosis. The cardiovascular-kidney-metabolic (CKM) syndrome construct (109) is complementary rather than competing: CKM describes a multisystem phenotype, and CASCADE resolves its atherosclerotic component into sequential phases. What CASCADE adds to these frameworks is the combination of phase-specific biomarker anchors, an explicit reversibility gradient, and vascular aging as a cross-cutting dimension.

### Convergence with contemporary calls for a richer prevention framework

A growing literature complements the chronological orientation proposed here by emphasizing that population-level risk models underestimate inter-individual variability in vascular response to identical exposures. Shapiro and Nasir (84) have argued that the prevention field has historically studied disease phenotypes while neglecting their counterpart — individuals who resist atherosclerosis despite substantial cumulative exposure — and have proposed vascular resilience as a second dimension deserving formal investigation. Akyol and colleagues (110) further document that LDL atherogenicity varies across subfractions and that proteoglycan-binding gradients modulate retention beyond particle concentration alone. The clinical relevance of this heterogeneity is reinforced by real-world data: in 9,446 statin-treated patients with well-controlled LDL-C following percutaneous coronary intervention, residual inflammatory risk remained the dominant predictor of recurrent events, independent of triglyceride levels and lipid burden (111). Recent commentary by preventive cardiologists has questioned whether particle exposure determines retention without consideration of endothelial conditions and matrix composition. CASCADE accommodates these positions within a single chronological framework, treating them as complementary observations of distinct temporal windows rather than as competing alternatives: heterogeneity in how individual vascular systems respond to a given cumulative exposure is precisely what the cross-cutting vascular-aging dimension is designed to capture.

### A two-dimensional architecture: risk exposure and vascular response

The framework proposed here can be read as an empirical operationalization of the two-dimensional structure that the contemporary prevention literature is converging upon. Along the horizontal axis, F0–F7 organize the trajectory of cumulative risk exposure. This axis captures what cumulative-exposure models — including the plaque-years formulation of Ference and colleagues — quantify across the lifespan. Along a cross-cutting axis, vascular aging organizes heterogeneity in how individual arterial systems respond to cumulative exposure: arterial stiffness, biological clocks, inflammatory tone, and structural integrity each contribute to the position a patient occupies independent of their phase. This corresponds to what Shapiro and Nasir term “vascular resilience” and to the residual variability that cumulative-exposure models acknowledge but do not resolve.

Empirical support for the two-axis architecture is accumulating. In a UK Biobank cohort of 34,092 individuals without prior cardiovascular disease followed for a median of 13.6 years, elevated Lp(a) was associated with major adverse cardiovascular events only in the stratum with elevated interleukin-6, not in the stratum with low IL-6 (interaction *p* = 0.008) (73). The signal was male-driven and attenuated at the clinically used threshold of ≥125 nmol/L, and IL-6 was measured on a relative platform scale rather than against validated clinical cut-offs — caveats that the authors themselves underscore. Within the framework proposed here, this interaction is interpretable as the empirical signature of two-dimensional risk: Lp(a) populates the horizontal axis as an F5 marker of cumulative exposure, IL-6 populates the cross-cutting axis as a marker of inflammaging, and the clinical event integrates both. CASCADE does not claim that this single observation validates the architecture; it claims that the architecture provides a structured way to ask whether analogous interactions exist for other phase–response pairs.

### Cardiovascular-kidney-metabolic (CKM) syndrome as integrative context

The 2023 AHA Presidential Advisory introduced the concept of CKM syndrome (109) to capture the interconnected pathophysiology of metabolic dysfunction, chronic kidney disease, and cardiovascular disease, and the 2026 ACC/AHA Multisociety Dyslipidemia Guideline formally incorporated CKM syndrome — with albuminuria, proteinuria, or chronic kidney disease as defining components — as a condition requiring intensified preventive intervention (17). CKM syndrome and CASCADE are complementary rather than overlapping. CKM syndrome defines a cross-sectional clinical phenotype: the simultaneous presence of metabolic dysfunction, kidney injury, and cardiovascular risk. CASCADE defines a longitudinal trajectory: the chronological sequence through which these features emerge, interact, and progress. The inclusion of albuminuria as a defining component of CKM syndrome provides independent clinical validation for the CASCADE decision to designate F3 as a distinct stage with UACR as its dominant biomarker, separable from the F4 window of atherogenic lipoprotein retention. In practical terms, a patient with CKM syndrome enters CASCADE at multiple phases simultaneously — typically F1, F3, and F4 — and the framework offers a structured way to identify which phase currently represents the most actionable intervention target.

## Limitations

CASCADE is a conceptual framework based on narrative synthesis of published evidence. Several limitations must be acknowledged explicitly.

First, the chronological sequence has not been demonstrated prospectively. No longitudinal study has followed patients from F0–F7 documenting sequential transitions. The proposed order represents the most complete pathophysiological trajectory supported by mechanistic evidence, but it is not the only trajectory. For instance, familial hypercholesterolemia or genetically elevated Lp(a) may enter the cascade at F4–F5 without preceding metabolic or environmental exposure. CASCADE presents the sequence as a testable hypothesis and acknowledges that phases may overlap temporally.

Second, the separation of endothelial injury and barrier dysfunction (F3) from atherogenic lipoprotein retention (F4) is the framework's most vulnerable architectural decision. As discussed in Section 1, the response-to-retention hypothesis treats subendothelial entry and retention as a mechanistic continuum. While CASCADE argues that albuminuria captures a clinically distinct pre-retention window, the temporal primacy of barrier dysfunction over retention has not been established in human atherosclerosis. Whether UACR and apoB identify distinct risk phenotypes is a testable question that prospective data could resolve.

Third, this framework has no outcome data of its own. It has not been validated in a prospective cohort, and the proposed biomarker assignments have not been tested as a composite panel against cardiovascular endpoints.

CASCADE is presented as a testable proposal, and its validation pathway can be specified. The natural first step is retrospective: applying the phase definitions to existing cohorts with stored biomarker and imaging data — including the cohorts underpinning PREVENT and SCORE2 — to establish the prevalence and co-occurrence of the proposed phases, and to test whether phase assignment improves calibration and discrimination (C-statistic), yields net reclassification improvement, or adds clinical net benefit on decision-curve analysis relative to the corresponding risk calculator. A second step is prospective: measuring the rates and temporal order of phase transitions, the reproducibility of phase assignment between observers, and the cost-effectiveness of any additional measurements the framework requires; and ultimately testing whether phase-guided intervention modifies clinically relevant outcomes. CASCADE is proposed with the explicit expectation of being tested — and revised — against these benchmarks.

Fourth, two of the eight proposed dominant biomarkers — the UPF-score (F0) and the integrated vascular age metric (F7) — lack external validation. The remaining six, while individually well-validated, have not been tested as a combined set. Their selection was pragmatic, guided by pathophysiological representativeness and clinical accessibility in a resource-stratified setting, not by statistical derivation. Alternative markers may prove superior for specific phases when evaluated in larger and more diverse populations. The depth of the supporting evidence is also uneven across phases: for F1, prospective cohorts associate insulin-resistance indices with incident cardiovascular events and mortality (112, 113), but no cohort has yet traced insulin resistance through the sequential vascular stages that CASCADE proposes, and F2 lacks dedicated incidence cohorts.

Fifth, the reversibility gradient (green/yellow/red) is proposed without quantitative thresholds for phase transitions. These thresholds require empirical derivation in prospective cohorts and will likely vary by population, age, and sex. Moreover, CASCADE assigns patients to discrete phases — a form of hard clustering — whereas evidence from precision medicine suggests that only approximately one-third of patients with type 2 diabetes can be classified into metabolic subtypes with 80% or greater certainty (114). The degree to which individuals occupy clear phases vs. transitional states remains an empirical question.

Sixth, vascular aging as a cross-cutting dimension shares the same validation gap: no study has prospectively evaluated whether measuring aging clocks at each phase improves risk prediction beyond what is achieved by the phase-specific biomarkers alone.

Seventh, the prevention field continues to debate whether atherosclerotic risk is best captured by cumulative concentration of apoB-containing particles or by kinetic parameters that incorporate particle residence time and oxidative modification. The cumulative-burden framing currently carries the largest base of prospective and trial-based evidence, including cohort data in which both cumulative LDL-cholesterol exposure and its time course independently predict incident event risk (115), while kinetic models offer mechanistic detail whose clinical translation is still developing; rather than choosing between them at this writing, CASCADE provides a chronological architecture in which kinetic, concentration, and inflammatory mechanisms coexist and can be located, compared, and tested at the phases where each operates. The empirical observation invoked in support of the two-dimensional architecture — the Lp(a) × IL-6 interaction reported in UK Biobank — derives from a single cohort with a male-driven signal, attenuation at the clinical Lp(a) threshold of ≥125 nmol/L, and IL-6 measured on a relative platform scale. It establishes feasibility of empirical interrogation of the framework rather than confirmation of any specific phase–response pair. Finally, large-scale population studies that anchor several of the proposed phases — including the eating-window distribution data informing F0 — derive from a self-selected cohort of myCircadianClock app users (*n* = 21,006), predominantly higher-educated English speakers, with portion sizes and total energy intake not captured (26). More broadly, the evidence base underpinning most phases derives predominantly from European and North American cohorts, so both the proposed sequence and its biomarker anchors may perform differently in other populations. Validation in populations less represented in the foundational evidence base, particularly Latin American cohorts, is therefore an explicit research priority.

Finally, CASCADE does not systematically integrate cardiovascular genetics — polygenic risk scores have demonstrated incremental reclassification value (116) but are not currently operationalizable in the clinical setting for which this framework was designed — and the psychosocial dimension is included as a contextual factor rather than a dedicated phase. Emerging evidence linking gut-derived metabolites — including trimethylamine N-oxide (TMAO) and imidazole propionate (ImP) — to both plaque biology and thrombotic risk suggests that the gut-vascular axis may warrant dedicated integration in future iterations of the framework. Similarly, metabolic dysfunction–associated steatotic liver disease (MASLD) has been identified as a cross-cutting amplifier of cardiovascular risk across multiple phases — from F1–F3 — with Fibrosis-4 index (FIB-4) ≥ 2.67 conferring a hazard ratio of 2.96 for cardiovascular mortality and meta-analyses of over 500,000 patients documenting a 64% increase in cardiovascular mortality (117). A dedicated evaluation of MASLD as a cross-cutting dimension, potentially parallel to vascular aging, is a priority for future iterations of the framework once prospective validation cohorts are available.

These limitations are structural features of a framework proposed as a hypothesis-generating contribution. CASCADE is intended to organize existing knowledge into a testable architecture, to identify gaps that existing risk tools do not address, and to invite empirical evaluation by the research community.

## Conclusion

Atherosclerosis is not a disease that begins with cholesterol and ends with a clinical event. It is a cascade that can originate in environmental factors and nutritional pressure, be amplified by psychosocial stress and mental health burden, progress through metabolic and vascular adaptation, cross an endothelial threshold, organize into atherogenic plaque, and manifest clinically — all within a vascular system that ages at its own pace.

CASCADE proposes a chronological framework with measurable biomarkers assigned to each phase, and vascular aging as a cross-cutting dimension that modulates the velocity and expression of every phase. The framework does not replace existing risk calculators but provides the organizational architecture within which they — and the growing body of evidence on insulin resistance, endothelial biology, plaque heterogeneity, and biological aging — can be contextualized. Its principal contribution is not a score but a structure: a way of asking where in the cascade a patient is, not merely how likely an event is.

Several of CASCADE's architectural decisions — particularly the separation of endothelial injury/barrier dysfunction from atherogenic lipoprotein retention, and the treatment of vascular aging as cross-cutting rather than sequential — are explicitly proposed as testable hypotheses rather than settled conclusions. The framework's value lies precisely in making these questions explicit and empirically addressable.

Advances in genomics, epigenomics, and biological aging research suggest that the predisposition toward specific cardiometabolic trajectories may be identifiable far earlier than current clinical practice assumes — potentially from the earliest stages of life. CASCADE proposes the border pieces of this puzzle: a chronological architecture that organizes the known phases and their measurable anchors. The center of the puzzle — prospective validation demonstrating that individuals traverse these phases in identifiable, modifiable patterns — remains to be assembled. This is the work that CASCADE invites.

## Data Availability

The original contributions presented in the study are included in the article/Supplementary Material, further inquiries can be directed to the corresponding author.
