# Supplementary material for: A chronological framework for atherosclerosis: from environmental pressure to clinical disease, with vascular aging as a cross-cutting dimension
Source: Front Cardiovasc Med. 2026 Oct 7;13:1941915. doi: 10.3389/fcvm.2026.1941915 (PMC13693078; doi:10.3389/fcvm.2026.1941915)
Supplement: Supplementary file 1 [file Table1.docx]

**Supplementary Table S1.** Evidence base of the CASCADE framework by phase and study design. (a) Number of cited references by phase and design level; (b) design level and phase assignment of every reference cited in the manuscript.

**(a) References by CASCADE phase and design level**

| **Phase** | **N1** | **N2** | **N3** | **N4** | **N5** | **N6** | **Total** |
| --- | --- | --- | --- | --- | --- | --- | --- |
| F0 · Environmental and behavioral pressure | 1 | 3 | 5 | 1 | 2 | 0 | 12 |
| F1 · Adaptive metabolic response | 3 | 2 | 1 | 2 | 4 | 0 | 12 |
| F2 · Vascular response and arterial stiffening | 1 | 3 | 1 | 2 | 2 | 0 | 9 |
| F3 · Endothelial injury and barrier dysfunction | 4 | 3 | 2 | 1 | 3 | 0 | 13 |
| F4 · Atherogenic lipoprotein retention | 3 | 2 | 1 | 0 | 7 | 0 | 13 |
| F5 · Plaque vulnerability and biological heterogeneity | 4 | 6 | 3 | 1 | 7 | 1 | 22 |
| F6 · Subclinical atherosclerosis | 0 | 4 | 3 | 0 | 2 | 1 | 10 |
| F7 · Clinical cardiovascular disease | 4 | 5 | 1 | 0 | 3 | 1 | 14 |
| EV · Vascular aging (cross-cutting) / general framework | 0 | 4 | 3 | 2 | 10 | 5 | 24 |

**(b) Design level and phase assignment of each cited reference**

| **Ref.** | **First author** | **Journal, year** | **Level** | **Phase(s)** |
| --- | --- | --- | --- | --- |
| 1 | D’Agostino RB Sr | Circulation 2008 | N2 | F7 |
| 2 | SCORE2 Working Group and ESC CVD Risk Collaboration | Eur Heart J 2021 | N2 | F7 |
| 3 | Khan SS | Circulation 2024 | N2 | F7 |
| 4 | Romeo S | Eur Heart J 2025 | N5 | F7 |
| 5 | Münzel T | Circulation 2026 | N5 | F0 |
| 6 | Shah VN | Lancet Diabetes Endocrinol 2026 | N6 | EV |
| 7 | Zaman S | Lancet 2025 | N5 | EV |
| 8 | Williams KJ | Arterioscler Thromb Vasc Biol 1995 | N5 | F3 |
| 9 | Borén J | Eur Heart J 2020 | N5 | F3, F4 |
| 10 | Horton WB | Am J Physiol Heart Circ Physiol 2025 | N5 | F1, F2 |
| 11 | Lichtenstein AH | Circulation 2026 | N5 | F0, F7 |
| 12 | Golaszewski NM | JAMA Netw Open 2022 | N2 | F0 |
| 13 | Yücel AD | Nat Rev Mol Cell Biol 2026 | N5 | EV |
| 14 | Spray L | Cardiovasc Res 2025 | N5 | EV |
| 15 | Evans PC | Eur Heart J 2026 | N5 | EV |
| 16 | Neumann JT | Eur Heart J 2025 | N5 | EV |
| 17 | Blumenthal RS | J Am Coll Cardiol 2026 | N5 | F4, F6 |
| 18 | Marfella R | N Engl J Med 2024 | N2 | F0 |
| 19 | Li J | Eur J Prev Cardiol 2025 | N2 | F0, F7 |
| 20 | Baumer Y | npj Cardiovasc Health 2025 | N3 | F0 |
| 21 | Mastrangelo A | Nature 2025 | N4 | F0 |
| 22 | Juraschek SP | Nat Med 2026 | N1 | F0 |
| 23 | GBD 2023 IHD & Dietary Risk Factors Collaborators | Nat Med 2026 | N3 | F0 |
| 24 | Patel CJ | Nat Med 2026 | N3 | F0 |
| 25 | Legaz A | Nat Med 2026 | N3 | F0 |
| 26 | Tran T | Nat Metab 2026 | N3 | F0 |
| 27 | Ma C | Sci Rep 2024 | N3 | F1 |
| 28 | Accili D | Nat Rev Endocrinol 2025 | N5 | F1 |
| 29 | Davies MJ | Nat Rev Dis Primers 2026 | N5 | F1 |
| 30 | Li J | Nat Commun 2026 | N4 | F1, F2 |
| 31 | Zhang T | Nat Cell Biol 2024 | N4 | F1 |
| 32 | Sethi S | Nat Mental Health 2026 | N5 | F1 |
| 33 | Deanfield J | Lancet 2025 | N1* | F1 |
| 34 | Reference Values for Arterial Stiffness' Collaboration | Eur Heart J 2010 | N3 | F2 |
| 35 | Greve SV | J Hypertens 2016 | N2 | F2 |
| 36 | Lagrange J | J Am Heart Assoc 2025 | N2 | F2 |
| 37 | Mitra R | Curr Atheroscler Rep 2017 | N5 | EV |
| 38 | Bar A | J Am Heart Assoc 2019 | N4 | F3 |
| 39 | Heerspink HJL | Ann Intern Med 2026 | N1 | F3 |
| 40 | Waijer SW | J Am Heart Assoc 2020 | N1* | F3 |
| 41 | Persson F | Diabetes Care 2021 | N1* | F3 |
| 42 | Zaitoon H | Nat Rev Nephrol 2026 | N5 | F3 |
| 43 | Palanca A | Atherosclerosis 2018 | N2 | F3 |
| 44 | Masrouri S | Eur J Prev Cardiol 2025 | N2 | F3 |
| 45 | Walendy V | Am J Prev Cardiol 2026 | N2 | F3 |
| 46 | Xuan Y | Diabetes Metab Syndr Obes 2026 | N3 | F3 |
| 47 | Shen FJ | Sleep Breath 2024 | N3 | F3 |
| 48 | Baier JM | Eur J Prev Cardiol 2025 | N1 | F3 |
| 49 | Döring Y | Nat Rev Cardiol 2024 | N5 | F4 |
| 50 | Mach F | Eur Heart J 2025 | N5 | F4, F6 |
| 51 | Peng AW | J Am Coll Cardiol 2025 | N2 | F4, F6 |
| 52 | Ference BA | Nat Rev Cardiol 2024 | N5 | F4 |
| 53 | Bohula EA | N Engl J Med 2026 | N1 | F4 |
| 54 | Marston NA | JAMA 2026 | N1 | F4 |
| 55 | Lee YJ | N Engl J Med 2026 | N1 | F4 |
| 56 | Di Fusco SA | High Blood Press Cardiovasc Prev 2025 | N5 | F4 |
| 57 | Tsimikas S | Nat Rev Cardiol 2024 | N5 | F5 |
| 58 | Erlinge D | J Am Coll Cardiol 2025 | N2 | F5 |
| 59 | Kronenberg F | Atherosclerosis 2025 | N5 | F5 |
| 60 | Reyes-Soffer G | Am J Prev Cardiol 2024 | N5 | F5 |
| 61 | Alonso-Herranz L | Nat Rev Cardiol 2026 | N6 | F5 |
| 62 | Mohammadi Jouabadi S | Eur J Prev Cardiol 2026 | N2 | F5 |
| 63 | Dong C | Nat Commun 2025 | N4 | F5 |
| 64 | Zhao F | Cell Mol Biol Lett 2025 | N5 | F5 |
| 65 | Barreto J | Arterioscler Thromb Vasc Biol 2021 | N5 | F5 |
| 66 | Kott KA | Biomolecules 2023 | N3 | F5 |
| 67 | Schiopu A | Ann Med 2023 | N3 | F5 |
| 68 | O’Donoghue ML | Nat Med 2025 | N1 | F5 |
| 69 | Fredman G | Nat Rev Cardiol 2024 | N5 | F5 |
| 70 | Ridker PM | N Engl J Med 2017 | N1 | F5 |
| 71 | Tardif JC | N Engl J Med 2019 | N1 | F5 |
| 72 | Nidorf SM | N Engl J Med 2020 | N1 | F5 |
| 73 | Bundgaard JS | Atherosclerosis 2026 | N2 | F5 |
| 74 | Wulff AB | Eur Heart J 2025 | N5 | F5 |
| 75 | Schulze K | Nat Rev Cardiol 2026 | N2 | F5 |
| 76 | Mazhar F | J Am Heart Assoc 2022 | N2 | F5 |
| 77 | Anindya K | Sci Rep 2026 | N3 | F6 |
| 78 | Yang X | Nat Commun 2025 | N3 | F6 |
| 79 | Bundgaard H | N Engl J Med 2026 | N2 | F6 |
| 80 | Zheutlin AR | Am J Med 2026 | N6 | F6 |
| 81 | Kang J | Lancet 2026 | N1 | F7 |
| 82 | Castellano JM | N Engl J Med 2022 | N1 | F7 |
| 83 | Colivicchi F | Eur Heart J Qual Care Clin Outcomes 2026 | N2 | F7 |
| 84 | Shapiro MD | Am J Prev Cardiol 2025 | N6 | F7 |
| 85 | Matteucci A | Eur J Haematol 2026 | N1 | F7 |
| 86 | Matteucci A | Open Heart 2025 | N1 | F7 |
| 87 | Matteucci A | Open Heart 2024 | N3 | F7 |
| 88 | Jaiswal S | N Engl J Med 2017 | N3 | EV |
| 89 | Kruger R | J Hypertens 2025 | N5 | EV |
| 90 | Bruno RM | Hypertension 2020 | N2 | F2, EV |
| 91 | Argentieri MA | Nat Med 2024 | N2 | EV |
| 92 | Sayed N | Nat Aging 2021 | N3 | EV |
| 93 | Gu K-N | Front Aging 2026 | N3 | EV |
| 94 | Vlachopoulos C | JAMA Netw Open 2019 | N1* | F2 |
| 95 | McEvoy JW | Eur Heart J 2024 | N5 | F2 |
| 97 | Yang Y | Cell 2024 | N4 | EV |
| 98 | Clayton ZS | Hypertension 2023 | N4 | F2, EV |
| 99 | Bakula D | Science 2026 | N6 | EV |
| 100 | Gómez Ortega J | bioRxiv (preprint) 2026 | N6 | EV |
| 101 | Bergmark BA | N Engl J Med 2025 | N1 | F1 |
| 102 | Marston NA | Circulation 2026 | N1 | F1 |
| 103 | Luebbe S | JAMA 2026 | N3 | F4 |
| 104 | Parini P | Curr Opin Lipidol 2026 | N5 | F4 |
| 105 | Kraaijenhof JM | Eur Heart J 2025 | N2 | F5 |
| 106 | Man S | J Am Coll Cardiol 2025 | N3 | F5, F6 |
| 107 | Sim M | JACC Adv 2026 | N2 | F6 |
| 108 | Larsen AF | Eur Heart J 2026 | N2 | F6 |
| 109 | Ndumele CE | Circulation 2023 | N5 | EV, F7 |
| 110 | Akyol O | Front Cardiovasc Med 2025 | N5 | EV |
| 111 | Di Muro FM | Eur J Prev Cardiol 2026 | N2 | EV |
| 112 | Lee JH | Arterioscler Thromb Vasc Biol 2023 | N2 | F1 |
| 113 | Li C | Clin Hypertens 2024 | N2 | F1 |
| 114 | Coral DE | Nat Med 2026 | N6 | EV |
| 115 | Domanski MJ | J Am Coll Cardiol 2020 | N2 | F4 |
| 116 | Busby GB | Nat Commun 2023 | N2 | EV |
| 117 | Ratti C | Atherosclerosis 2025 | N5 | EV |
| 118 | Montoya-Pérez JE | Preprints.org 2026 | N6 | EV |

**Design levels:** N1, randomized controlled trial, meta-analysis, or individual-participant-data analysis; N2, prospective cohort or registry; N3, cross-sectional, case-control, or modelling study; N4, mechanistic or experimental study; N5, guideline, consensus statement, or narrative review; N6, preprint, editorial, or commentary. N1* denotes a secondary or post hoc analysis of a randomized trial. EV denotes references supporting vascular aging as a cross-cutting dimension or the general framework rather than a single phase. A reference assigned to more than one phase is counted once in each phase in (a) and listed once in (b). Design level was assigned by the authors from the study design stated in each publication; it is a descriptive classification of study type, not a formal certainty-of-evidence grading (e.g., GRADE). Phase names follow Section 1 of the main text.
